# Investigating the Role of TRPV4 and GPR35 Interaction in Endothelial Dysfunction in Aging Mice

**DOI:** 10.1111/acel.14469

**Published:** 2025-01-02

**Authors:** Xiaoxue Tian, Hao Kan, Liu Yang, Zhiwei Wang, Tiantian Zhang, Ka Zhang, Aiqin Mao, Xin Wen, Tingting Zhou, Xiaoyan Wang, Xiaodong Zhang, Lei Feng, Li Geng

**Affiliations:** ^1^ Wuxi School of Medicine Jiangnan University Wuxi China; ^2^ Department of Cardiology The Affiliated Hospital of Jiangnan University Wuxi China

**Keywords:** aging, calcium, endothelial cell, endothelial dysfunction, GPR35, protein–protein interaction, TRPV4, vasodilation

## Abstract

Endothelial dysfunction, characterized by a decline in endothelial physiological functions, is a significant aspect of cardiovascular aging, contributing notably to arterial stiffness, atherosclerosis, and hypertension. Transient receptor potential channel V4 (TRPV4), a key member of Ca^2+^‐permeable channels, plays a crucial role in maintaining vascular functions. However, the role and mechanisms of TRPV4 in aging‐related endothelial dysfunction remain incompletely understood. Here, we demonstrated a marked reduction in endothelial TRPV4 function without alterations in its expression, leading to abnormal endothelial Ca^2+^ signaling and impaired vasodilation in aging mesenteric arteries. Employing transcriptome sequencing, co‐IP, and PLA assays, we characterized G protein‐coupled receptor 35 (GPR35) interacting with TRPV4, and abnormally enhanced interactions were found in aging endothelial cells. Subsequently, we revealed that intensive GPR35‐TRPV4 interaction significantly contributes to endothelial dysfunction during aging, utilizing TRPV4 endothelial‐specific knockout (TRPV4_EC_^−/−^), AAV‐FLT1‐shRNA (GPR35) mice, and GPR35 overexpressed/knocked‐down HUVECs. Furthermore, molecular docking analysis and subsequent co‐IP and pressure myograph experiments indicated that both Thonningianin A and Carfilzomib efficiently restored the GPR35‐TRPV4 interaction, preventing endothelial dysfunction and vasodilation impairment. Our study identifies the crucial role of GPR35‐TRPV4 interaction in aging‐associated abnormal endothelial function and vascular tone modulation. Restoring GPR35‐TRPV4 interaction via Thonningianin A or Carfilzomib represents a promising precision approach for aging‐related endothelial dysfunction.

## Graphical abstract

The diminished function of endothelial TRPV4, rather than changes in its expression, plays a significant role in aging‐related endothelial dysfunction. The interaction between TRPV4 and GPR35 significantly contributes to this condition. Blocking the TRPV4‐GPR35 interaction with Thonningianin A or Carfilzomib represents a promising precision approach for treating aging‐related endothelial dysfunction.

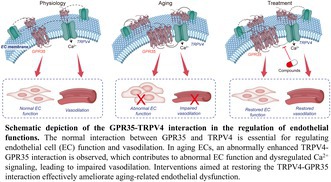

## 1 Introduction

The endothelium, a monolayer of cells lining the inner surface of blood vessels, plays an active role in preserving vascular integrity (Rajendran et al. 2013; Xu et al. 2021). A fundamental role it plays is in regulating vascular tone, thereby facilitating blood vessel relaxation, optimizing blood flow, and preventing hypertension (Sandoo et al. 2010). Various risk factors including aging, obesity, and diabetes can induce changes in endothelial morphology and function, contributing to arterial stiffness, atherosclerosis, hypertension, stroke, and coronary artery disease (Jia et al. 2019; Kresnajati et al. 2022; Powell‐Wiley et al. 2021). Particularly in aging, endothelial dysfunction emerges as a significant aspect of cardiovascular aging, characterized by a decline in endothelial physiological functions (Donato, Machin, and Lesniewski 2018; Han and Kim 2023; Seals, Jablonski, and Donato 2011). As individuals age, endothelial cells (ECs) undergo alterations that impair their capacity to regulate vascular tone and maintain homeostasis. Factors such as oxidative stress, inflammation, and reduced endothelial repair mechanisms further exacerbate endothelial dysfunction with age (Donato et al. 2007; Higashi 2022). This dysfunction plays a central role in the onset and progression of age‐related cardiovascular conditions like atherosclerosis, hypertension, and heart failure. Therefore, comprehending the mechanisms driving aging‐related endothelial dysfunction is vital for developing interventions that could attenuate its impact and promote cardiovascular health among the elderly population.

Transient receptor potential (TRP) channels have emerged as important regulators of arterial functions (Earley and Brayden 2015). Recent studies have solidified the pivotal role of TRPV4 channels in modulating vascular reactivity (Chen and Sonkusare 2020; Ottolini, Hong, and Sonkusare 2019). Particularly, EC TRPV4 channels are widely acknowledged as facilitators of vasodilation, a process integral to reducing vascular resistance (Sonkusare et al. 2012, 2014). Notably, endothelial TRPV4 channels are known to form functional complexes with various proteins across different vascular beds, thereby exerting significant influences on diverse cardiovascular processes. The interaction between TRPV4 and KCa3.1 channels is key in regulating coronary vascular tone, with disruptions linked to coronary artery disease (Mao et al. 2023). Moreover, impaired TRPV4–eNOS interaction in aortic arteries is identified as a significant factor in hypertension progression (Mao et al. 2022). In diet‐induced obesity, dysfunction of the AKAP150_EC_‐TRPV4_EC_ channel correlates with elevated blood pressure (Ottolini et al. 2020). Additionally, impaired endothelial signaling through the pannexin 1‐TRPV4 channel is strongly associated with pulmonary hypertension (Daneva et al. 2021). However, the specific role of TRPV4 in aging‐related endothelial dysfunction warrants further elucidation.

In the current study, we utilized littermates of age‐matched wild‐type (WT) and TRPV4 endothelial specific knockout (TRPV4_EC_^−/−^) mice at both young (3–4 months; equivalent to humans aged 20–30 years) and aging (20–24 months; equivalent to humans aged 70–90 years) stages to investigate the specific involvement of TRPV4 in age‐related endothelial dysfunction. Additionally, we employed transcriptome sequencing to comprehensively characterize G protein‐coupled receptor 35 (GPR35), a membrane‐bound protein interacting with TRPV4 to mediate endothelial functions. Our findings revealed that the abnormally heightened GPR35‐TRPV4 interaction significantly contributes to endothelial dysfunction during aging, as evidenced both in vitro and in vivo. Moreover, restoring the GPR35‐TRPV4 interaction using Thonningianin A or Carfilzomib effectively prevented endothelial dysfunction and impaired vasodilation in small mesenteric arteries of aging mice. Collectively, our study unveils, for the first time, the regulatory role of endothelial GPR35‐TRPV4 interaction in endothelial functions. Targeting the GPR35‐TRPV4 complex with selective compounds may offer a promising therapeutic approach for addressing impaired vasodilation in aging.

## 2 Materials and Methods

### 2.1 Reagents

GSK1016790A (GSK101, G0798), HC067047 (HC0, 616521), Zaprinast (Z0878), anti‐GPR35 antibodies (SAB4501277), and Duolink proximity ligation assay (PLA) kit (DUO96010) were procured from Sigma‐Aldrich (St. Louis, USA). Fluo‐4/AM (F14217) was acquired from Invitrogen (Carlsbad, USA). Anti‐TRPV4 antibodies from rabbit (ACC‐034) were sourced from Alomone Labs (Jerusalem, Israel), while anti‐TRPV4 antibodies from mouse were from Sigma‐Aldrich (MABS466). Normal rabbit IgG (AC005), utilized as a negative control for immunoprecipitation, was obtained from Abclonal (Wuhan, China). Carfilzomib (C795940), Thonningianin A (T873539), and naringin (767903) were obtained from Macklin (Shanghai, China), whereas Navitoclax (HY‐10087) and Diosmin (HY‐N0178) was sourced from MedChemExpress (Shanghai, China). All other reagents and chemicals were procured from Sigma‐Aldrich unless specified otherwise.

### 2.2 Animals

Endothelium‐specific TRPV4 knockout (TRPV4_EC_^−/−^) mice were generated by crossing TRPV4^flox/flox^ mice (Strain NO. T010226, GemPharmatech, Nanjing, China) with Tie2Cre^ERT2^ mice (JAX:030597). Specifically, 4‐week‐old Tie2Cre^ERT2+/0^; TRPV4^flox/flox^ mice and control littermates (TRPV4^flox/flox^) underwent a 10‐day treatment regimen of daily intraperitoneal injections of tamoxifen (40 mg/kg per day, diluted in corn oil) (Moore et al. 2013; Spiranec et al. 2018). After a 2‐week washout period and genotype confirmation, male mice were selected for experiments. TRPV4‐eGFP mice were obtained from the Mutant Mouse Regional Resource Centers. Aging models were established by randomly grouping male mice into young (3–4 months) and aging (20–24 months) categories for experimentation. All animals were housed under standard conditions at Jiangnan University, maintained on a 12‐h light/12‐h dark cycle, and provided with ad libitum access to food and water. Mice were euthanized by CO_2_ inhalation in combination with cervical dislocation. All animal experiments conformed to the Guide for the Care and Use of Laboratory Animals published by the US National Institutes of Health and were approved by Jiangnan University Animal Experimentations Ethics Committee (approval number: JN.No20201030t0221010[263]).

### 2.3 Isolation of Primary ECs

Third‐order mesenteric arteries were utilized throughout the study. Freshly isolated ECs from these arteries were used without passaging, following established protocols. In brief, the mesenteric vessels were carefully dissected by removing the mesenteric bed along the small intestine. After eliminating venous branches, the remaining arterial branches underwent digestion with 0.02% collagenase type IA for 45 min at 37°C. Subsequently, the pelleted cells were centrifuged at 1600 g for 5 min, and the resulting cell pellets were resuspended in EC growth medium (Lonza, Basel, Switzerland) supplemented with 10% fetal bovine serum (FBS). The medium was refreshed after 2 h, and the remaining ECs were cultured at 37°C with 5% CO_2_.

### 2.4 Intracellular Ca^2+^ Measurement

Primary ECs were incubated with 10 μM Fluo‐4/AM and 0.02% pluronic F‐127 for 30 min in the absence of light at 37°C. Subsequently, the changes in intracellular calcium concentration ([Ca^2+^]_i_) induced by GSK101 or Zaprinast were assessed. Fluo‐4 fluorescence was recorded using excitation at 488 nm with a confocal microscope (Zeiss LSM 880, Zeiss Microscopy, Germany). The *F*_*1*_/*F*_*0*_ values were determined by dividing the fluorescence of active sites in the region of interest (ROI) by the basal fluorescence observed over the measurement period.

### 2.5 Whole‐Cell Patch Clamp

Whole‐cell currents were recorded using conventional whole‐cell configurations employing an EPC10 patch clamp amplifier (HEKA, Holliston, USA) as described previously (He et al. 2017; Ma et al. 2010). All cells were utilized immediately after isolation. To assess TRPV4 currents, the pipette solution comprised 20 mM CsCl, 100 mM Cs^+^‐aspartate, 1 mM MgCl_2_, 4 mM ATP, 0.08 mM CaCl_2_, 10 mM BAPTA, 10 mM HEPES, pH 7.2. The bath solution contained 150 mM NaCl, 6 mM CsCl, 1 mM MgCl_2_, 1.5 mM CaCl_2_, 10 mM glucose, 10 mM HEPES, pH 7.4. Cs^+^ was introduced to mitigate the influence of K^+^ channels. Experiments were conducted at room temperature. All inhibitors were added to the bath solutions and pre‐incubated with cells before applications of agonists, if necessary. Recordings were obtained using a 500 ms voltage ramp protocol from −100 mV to +100 mV (Patchmaster; HEKA).

### 2.6 Wire Myography

Mesenteric arteries were isolated, cut into 2‐mm rings, and placed into a chamber filled with Krebs solution. The artery's activity was maintained by bubbling 95% O_2_/5% CO_2_ and maintaining a temperature of 37°C. After allowing the rings to equilibrate at baseline tension (2 mN) for 1 h, 60 mM KCl was applied to confirm arterial activity. The rings were then contracted using 1 μM of phenylephrine (Phe). Once the tension stabilized, agonists were added to observe changes in vascular tension.

### 2.7 Pressure Myography

Third‐order mesenteric arteries were isolated without surrounding tissues and mounted on glass micropipettes within a chamber (Danish Myo Technology, Aarhus, Denmark). Consistent cannulating pipettes with an inner diameter of 0.86 mm were utilized for all experiments. Prior to experimentation, the arteries were gradually pressurized to 80 mmHg in 37°C Krebs solution bubbled with 95% O_2_ and 5% CO_2_ to ensure proper vessel function. The external diameter was continuously monitored using MyoView software (Photonics Engineering, Hinnerup, Denmark) coupled with an inverted microscope (Axiovert 40; Zeiss, Oberkochen, Germany) equipped with a CCD camera module. Precontraction of all arteries was achieved by applying 1 μM Phe at 60 mmHg intraluminal pressure. Phe was dissolved in deionized water and diluted in Krebs solution. Flow adjustments were made by modifying the pressure of the inflow and outflow, establishing a pressure differential between the two ends of the arteries to achieve the target flow rate indicated in the software without altering intravascular pressure (Mao et al. 2022; Zhu et al. 2021). Maximal passive dilation was initiated by replacing the Ca^2+^‐free Krebs solution at the conclusion of each measurement. In select experiments, endothelial denudation was performed by passing a large air bubble through the arteries for 30 s. The loss of endothelial function was confirmed by the absence of a dilation response to 1 μM NS309 and 100 nM bradykinin. Arterial dilation was quantified as a percentage of the difference in diameter between the induced dilation and maximal dilation.

### 2.8 Bulk RNA‐Seq Analysis

Primary ECs isolated from third‐order mesenteric arteries were subjected to RNA isolation using MagZol Reagent. Subsequently, libraries were sequenced using Illumina novaseq 6000, resulting in an average of 41.4 million reads per sample (paired end, 2 × 150 bp). Quality control and adapter read trimming were conducted using Cutadapt (version 1.9.1). Gene quantification, including read count and normalized expression values as Fragments Per Kilobase Million (FPKM), was performed using Hisat2 version 2.0.1, based on the GENCODE GRCm38 genome. Differential expression analysis was carried out using DESeq2 version 1.26.0. We applied a threshold of an absolute Fold Change of ≥ 1.5 and an adjusted *p*‐value < 0.05 to identify significant changes between two conditions. Additionally, we enforced an expression value threshold of at least 2 FPKM in 2 samples. The expression patterns of selected genes were visualized as a heatmap of the *z*‐score of the Log_10_(FPKM + 1) using the pheatmap package version 1.0.12.

### 2.9 Western Blot

All cells were used after isolation without undergoing passages. Each isolation of third‐order mesenteric ECs involves the use of 3 mice. The samples underwent lysis using lysis buffer, and the lysates were subsequently collected by centrifugation. Protein samples were then separated via 10% SDS gel electrophoresis and transferred onto an immobilon‐P polyvinylidene difluoride membrane (Millipore Corp., Bedford, USA). Following this, the membranes were blocked with 5% BSA and incubated overnight at 4°C with primary antibodies against TRPV4 (1:500), GPR35 (1:500), GAPDH (1:1000) and Tubulin (1:1000), followed by incubation with HRP‐conjugated secondary antibodies. Band intensity analysis was performed using ImageJ software.

### 2.10 Real‐Time qPCR

Total RNA extracted was isolated using MagZol Reagent, and then reverse‐transcribed into cDNA using an appropriate kit (Yeasen, Shanghai, China). Real‐time quantitative PCR (RT‐qPCR) reactions were conducted using SYBR Green Master Mix (Yeasen, Shanghai, China). The thermocycling conditions were as follows: initial denaturation at 95°C for 30 s, followed by 40 cycles of denaturation at 95°C for 5 s and annealing/extension at 60°C for 30 s, utilizing a real‐time PCR cycler (Roche Light Cycler 480 II, Basel, Switzerland). The relative expression level of the target mRNA was determined using the 2^−ΔΔCt^ method, with GAPDH expression serving as the internal control (Zheng et al. 2021).

### 2.11 Proximity Ligation Assay

ECs were fixed with 4% paraformaldehyde (PFA) for 30 min, followed by three washes with PBS. Subsequently, the cells were permeabilized with 0.1% Triton X‐100 for 30 min at room temperature, and then blocked with 5% bovine serum albumin (BSA) and 300 mM glycine for 1 h at room temperature. After three additional washes with PBS, the cells were incubated with primary antibodies overnight at 4°C. The PLA assay protocol from the Duolink PLA kit was then followed to detect co‐localized proteins. Finally, the cells were stained with 0.3 μmol/L DAPI for 10 min at room temperature in the dark. PLA images were captured using the Zeiss confocal imaging system. Image analysis involved normalizing the number of positive puncta by the number of nuclei in a given field of view. The specificity of the PLA antibodies was confirmed using ECs obtained from endothelial knockout mice for one of the protein pairs.

### 2.12 Co‐Immunoprecipitation

Immunoprecipitation experiments were conducted following standard protocols. Each isolation of primary EC samples uses mesenteric arteries from 5 mice. All ECs were harvested after isolation and cultured until reaching confluence without undergoing passages. Samples were lysed using immunoprecipitation (IP) buffer containing 50 mmol/L Tris–HCl (pH 7.4), 150 mmol/L NaCl, 0.3% NP‐40, and 2 mmol/L EDTA, supplemented with freshly prepared protease inhibitor cocktail. The extracted protein was then incubated with anti‐GPR35 antibody or species‐matched negative control IgG overnight at 4°C. Subsequently, the protein‐antibody complex was immunoprecipitated using protein A/G immunoprecipitation magnetic beads (Selleck, China) for an additional 4 h. The precipitated complexes were washed five times with IP buffer, eluted with 2× SDS loading buffer, and separated by SDS‐PAGE, followed by immunoblotting (IB) using anti‐TRPV4 antibody.

### 2.13 Plasmid Transfection

The cDNA fragments that encoded full‐length mouse GPR35 (NCBI reference sequence: NM_001104529.2) were cloned and inserted into the pcDNA3.1 vector plasmid. Cells cultured in 6‐well plates were transfected with 2 μg plasmids per well using Lipofectamine 3000 Reagent (Invitrogen, CA, USA).

### 2.14 siRNA Interfering

Small interfering RNAs (siRNAs) against mouse GPR35 were designed by GenePharma (Suzhou, China). Human umbilical vein endothelial cells (HUVECs) were from Cell Resource Center of Shanghai Biological Sciences Institute, Chinese Academy of Sciences, Shanghai, China, and used passages 3–6 throughout the study. Cells were cultured in 6‐well plates and transfected with 25 nmol/L siRNA per well using Lipofectamine 3000 Reagent (Figure S1). The siRNA sequences were as follows: GPR35 sense: GCUUCCGUCAACAACUUCUTT; GPR35 antisense: AGAAGUUGUUGACGGAAGCTT.

### 2.15 Endothelial Cell‐Specific Adeno‐Associated Viruses

To generate EC‐specific adeno‐associated viruses (AAVs), the GPR35 genes were subcloned into pAOV.SYN.mScarlet.3FLAG plasmids to create pAOV.SYN.mScarlet.GPR35.3FLAG (OBiO Technology, Shanghai, China), which contained an EC‐specific promoter, FLT1. AAV‐FLT1‐shRNA (GPR35) was generated by transfecting AAV‐293 cells with pAOV.SYN.mScarlet.GPR35.3FLAG along with AAV helper plasmid (pAAV Helper) and AAV Rep/Cap expression plasmid. AAV‐FLT1‐mScarlet‐shRNA (NC) was also prepared as a control. Viral particles were purified via iodixanol step‐gradient ultracentrifugation. The genomic titer was determined to be 2.5–3.5 × 10^12^ genomic copies per ml using quantitative PCR. AAV‐FLT1‐shRNA (GPR35) and AAV‐FLT1‐shRNA (NC) were administered to mice via tail vein injection, and their effects were assessed after 21 days.

### 2.16 Migration Assay

Cells were detached and suspended in serum‐free medium. A total of 3 × 10^4^ cells were seeded into the upper chamber of transwell inserts. The inserts were then placed into the lower chamber of a 24‐well plate containing medium supplemented with 10% FBS. After incubation for 24 h, non‐migrated cells on the upper side of the insert membrane were gently removed using wet cotton swabs. The migrated cells on the lower side of the membrane were fixed with 4% PFA and stained with 0.1% crystal violet. The number of migrated cells was counted in 9–12 randomly selected fields.

### 2.17 Tube Formation Assay

Matrigel matrix (BD Biosciences, Oxford, UK) with reduced growth factors was thawed overnight at 4°C. Prior to use, 96‐well plates and tips were chilled at 4°C for at least 2 h. Matrigel was added to the wells and allowed to solidify at 37°C for 30–45 min. Subsequently, 3 × 10^4^ ECs were seeded onto the Matrigel‐coated plates and incubated at 37°C for 4 h. A hypoxic environment was created in a humidified multi‐gas incubator with 1% O_2_, 5% CO_2_, and 94% N_2_ (Thermo, MA, USA). Images of the formed networks were captured using a microscope‐mounted camera (Nikon Coolpix 54, Tokyo, Japan). Quantitative analysis was performed on five randomly selected regions per well to evaluate the extent of tube formation. Total branch length, representing the cumulative length of vessel branches formed between junctions, was measured to assess the extent of branching and network complexity. Additionally, total branching length, which combines total segment length and total branch length, was calculated to reflect the overall size of the formed vascular network.

### 2.18 Cell Counting Kit‐8 (CCK‐8) Assay

Cells were seeded in 96‐well plates at a density of 2000 cells per well in medium 1 day prior to the experiment. Cell viability was assessed using the CCK‐8 kit (Yeasen, Shanghai, China) according to the manufacturer's instructions.

### 2.19 Molecular Docking

The crystal structures of TRPV4 (PDB accession number 4DX1) and GPR35 (PDB accession number AF_AFQ9ES90F1) proteins were obtained from the Protein Data Bank (PDB) website. ZDOCK docking was conducted using BIOVIA's Discovery Studio 2018 software to predict the interaction between TRPV4 and GPR35. Virtual amino acid mutations were introduced to the GPR35‐TRPV4 complex to identify key amino acids in the active site. Compounds with the highest affinity for reducing the GPR35‐TRPV4 interaction were then screened using LibDock.

### 2.20 Statistical Analysis

The data are presented as mean ± standard deviation (SD). Statistical analyses were conducted using GraphPad Prism 8.0 software (GraphPad Software, San Diego, CA, USA). Initially, the Shapiro–Wilks test was employed to assess the normal distribution of the data. For two‐group comparisons with normally distributed data and similar variances, Student's *t*‐test was utilized. In cases where equal standard deviations were not assumed, Welch's correction was applied. When comparing more than two groups, the Brown–Forsythe test was used to evaluate similar variances. Subsequently, ordinary ANOVA or Welch ANOVA tests were performed based on whether similar variances were assumed. A significance level of *p* < 0.05 was considered statistically significant. The specific statistical methods applied to each experiment are described in the corresponding figure legends.

## 3 Results

### 3.1 Loss of TRPV4 Function Is Associated With Impaired Endothelial Ca^2+^ Signaling and Vasodilation in Aging Mesenteric Arteries

**FIGURE 1:**
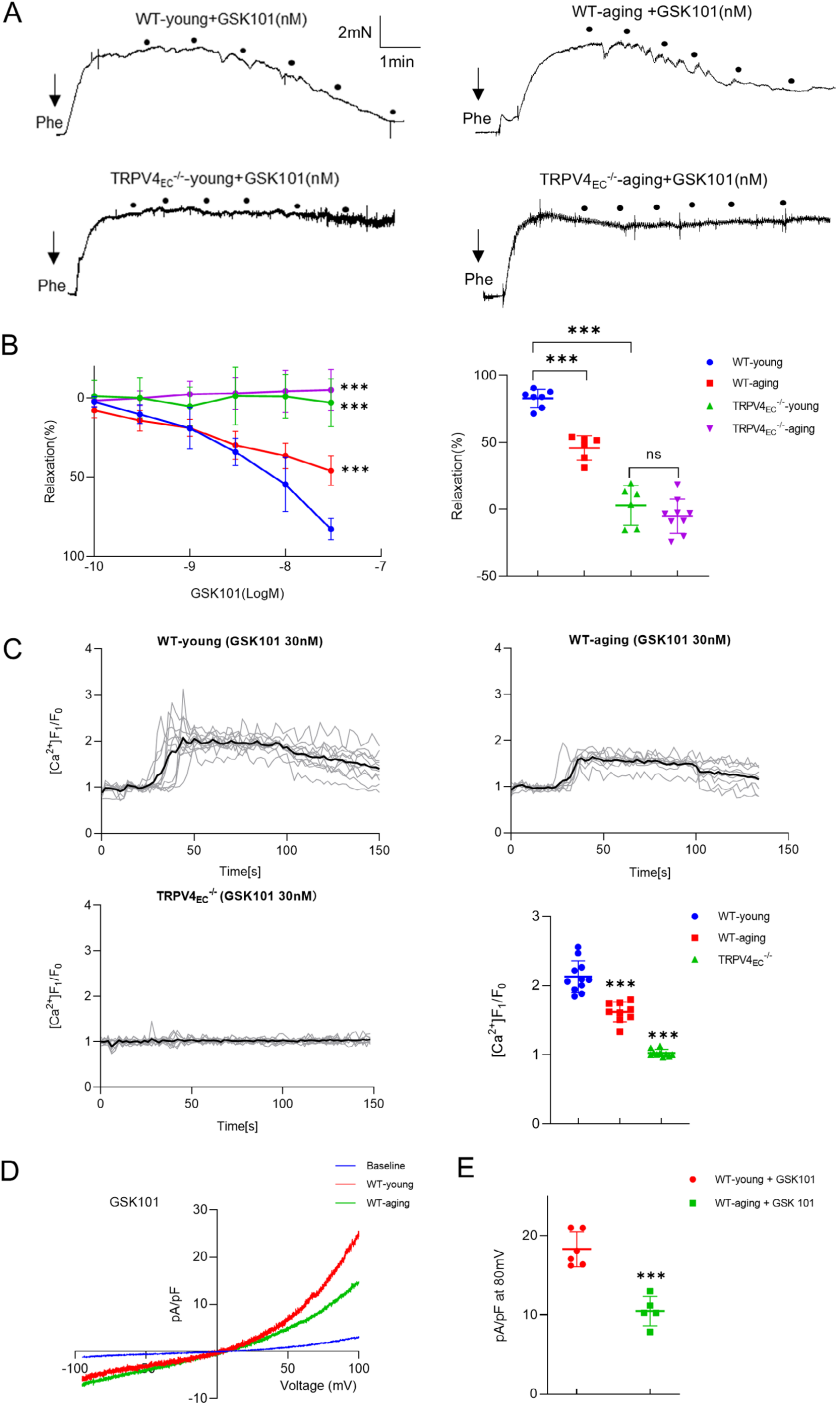
The impaired function of TRPV4 channels in primary ECs of aging mesenteric arteries. (A) Representative traces depicting gradient concentrations of GSK101‐induced vasodilation in mesenteric arteries from WT‐young (top left), WT‐aging (top right), TRPV4_EC_^−/−^‐young (bottom left), and TRPV4_EC_^−/−^‐aging (bottom right) groups. (B) Left panel: The dose‐dependent of GSK101‐induced vasorelaxation of mesenteric arteries from the groups listed in (A); right panel: The quantification of the maximal vessel relaxation percentages in the presence of GSK101 from the groups listed in (A) (*n* = 6–9 arteries for each group; ***p < 0.001 vs. WT‐young mice; two‐way ANOVA). (C) Representative traces and summarized data showing the time course of GSK101 (30 nM)‐induced Ca^2+^ influx in primary mesenteric ECs from WT‐young, WT‐aging, and TRPV4_EC_^−/−^ mice (*n* = 10–12 fields from 5 to 6 isolations for each group; ***p < 0.001 vs. WT young ECs; one‐way ANOVA). (D) Representative traces of TRPV4 channel currents from whole‐cell patch clamp recordings of isolated WT‐young (top) and WT‐aging (bottom) ECs challenged with GSK101 (30 nM). (E) Comparisons of the TRPV4 channel current densities at 80 mV between groups from (A) (*n* = 5 to 6 cells from 3 isolations for each group; ***p < 0.001 vs. WT‐young ECs as indicated in the figure; *t*‐test).

It is well established that aging is associated with a decline in endothelial function and vasodilation across various arterial beds (Brandes, Fleming, and Busse 2005; Ungvari et al. 2018). To explore whether endothelial TRPV4 contributes to the reduction in aging‐related vasodilation in small mesenteric arteries, myography experiments were conducted. A significant decrease in relaxation was observed in GSK101‐induced vasodilation in aging mesenteric arteries compared to WT‐young controls (Figure 1A,B). Notably, neither young nor aging TRPV4^−/−^ mesenteric arteries exhibited relaxation when stimulated with GSK101. To further explore the mechanism underlying TRPV4‐associated impaired vasodilation, primary ECs from mesenteric arteries were used. Endothelial identity was confirmed (Figure S2). Western blotting and imaging analyses revealed no significant differences in TRPV4 expression between WT‐young and WT‐aging groups (Figure S3). However, GSK101‐induced Ca^2+^ influx was significantly lower in primary ECs from aging mesenteric arteries compared to young mesenteric arteries (Figure 1C). This reduction in Ca^2+^ influx was accompanied by a marked decrease in TRPV4 currents in aging ECs compared to young ECs, as demonstrated by whole‐cell patch clamp experiments (Figure 1D,E). These findings collectively suggest that changes in TRPV4 function, rather than TRPV4 protein expression, may contribute to impaired vasodilation in aging mesenteric arteries.

### 3.2 Enhanced Interaction Between TRPV4 and GPR35 Was Observed in Aging Mesenteric Arteries

**FIGURE 2:**
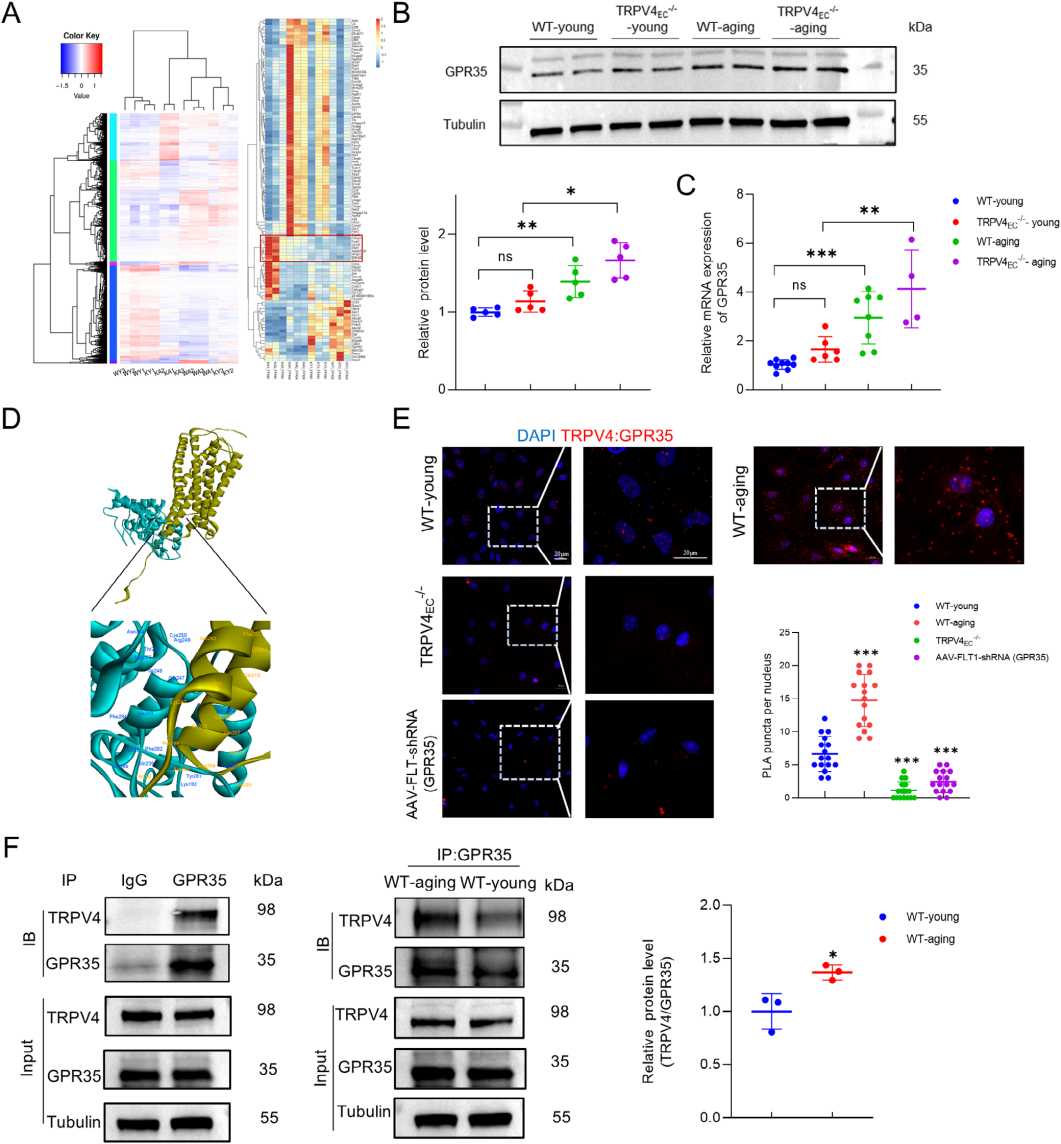
Enhanced GPR35‐TRPV4 interaction in primary mesenteric ECs during aging. (A) Left panel: Differential gene clustering map. Clustering was performed based on log_10_(FPKM + 1) values, with red indicating high expression genes and blue indicating low expression genes. KA, TRPV4_EC_^−/−^‐aging; KY, TRPV4_EC_^−/−^‐young; WA, WT‐aging; WY, WT‐young; Right panel: Genes that exhibit significant differences when comparing at least WY vs. WA and WA vs. KA. (B) Western blots of GPR35 expression in primary mesenteric ECs from all groups listed in (A); *n* = 5 isolations for each group; ns indicates no statistical significance when comparing with WT‐young ECs; **p < 0.01 vs. WT‐young ECs; *p < 0.05 vs. WT‐aging ECs; two‐way ANOVA. (C) Relative mRNA expression of GPR35 in primary mesenteric ECs from all groups listed in (A); *n* = 4–9 isolations for each group; ns indicates no statistical significance when comparing with WT‐young ECs; ***p < 0.001 vs. WT‐young ECs; **p < 0.01 vs. WT‐aging ECs; two‐way ANOVA. (D) Molecular docking of the 3D structures of TRPV4 (green) and GPR35 (yellow) with predicted binding sites. (E) Representative merged images of proximity ligation assays (PLAs) signal, showing EC nuclei and TRPV4_EC_‐GPR35_EC_ co‐localization (red puncta) in primary ECs from third‐order mesenteric arteries from WT‐young, WT‐aging, TRPV4_EC_^−/−^ and AAV‐FLT‐shRNA (GPR35) mice. Scale bar: 20 μm. Right panel: Quantification of TRPV4_EC_‐GPR35_EC_ co‐localization (*n* = 16 cells from 4 to 5 isolations for each group; ***p < 0.001 vs. WT‐young group; one‐way ANOVA). (F) Co‐immunoprecipitation (co‐IP) assay of TRPV4 and GPR35 in primary ECs from WT‐young and WT‐aging third‐order mesenteric arteries. The lysates were immunoprecipitated with control IgG or GPR35 antibody, followed by immunoblotting (IB) with TRPV4 antibody. The quantitative analysis is shown on the right panel (*n* = 3 isolations for each group; *p < 0.05 vs. WT‐young ECs; *t*‐test).

To further elucidate the mechanisms involving TRPV4 in regulating endothelial functions, primary ECs isolated from third‐order mesenteric arteries of WT‐young, WT‐aging, TRPV4_EC_^−/−^‐young and TRPV4_EC_^−/−^‐aging mice were subjected for RNA sequencing. The differentially expressed genes are depicted in Figure S4, comparing WT‐aging vs. WT‐young, TRPV4_EC_^−/−^‐young vs. WT‐young, TRPV4_EC_^−/−^‐young vs. TRPV4_EC_^−/−^‐aging, and WT‐aging vs. TRPV4_EC_^−/−^‐aging, respectively. The objective of the RNA‐seq analysis is to identify potential proteins that may interact with TRPV4 to modulate endothelial functions (Figure 2A). Further analysis revealed candidate genes with elevated expression levels in both aging and TRPV4_EC_^−/−^ groups compared to WT controls. The most significantly different genes are listed in Table S1. Among these candidates, GPR35 emerged as the most relevant membrane protein based on existing literature, owing to its documented role in endothelial functions (Li et al. 2021). Subsequently, we validated the significantly increased expression of GPR35 in WT‐aging and TRPV4_EC_^−/−^ groups compared to WT controls through Western blotting and RT‐qPCR analysis (Figure 2B,C). Next, we performed ZDOCK analysis to predict sites of protein recognition and interaction. The docking configuration with the highest ZDOCK score was selected for subsequent RDOCK optimization. Figure 2D illustrates the binding of TRPV4's anchoring domain to the intracellular domain of GPCR35. Afterward, Calculate Mutation Energy (Binding) was utilized to conduct interaction‐based virtual amino acid mutations on the TRPV4‐GPCR35 protein complex, identifying key amino acids in the active sites. The Table S2 lists the most probable binding sites involved in the interaction, based on the mutation energy scores. Endothelial TRPV4 often forms complexes with other proteins to exert its function in the vascular system (He et al. 2017; Mao et al. 2022). To investigate whether TRPV4 directly interacts with GPR35 in primary cultured ECs, PLA and co‐immunoprecipitation were performed (Figure 2E,F). These assays revealed a physical interaction between TRPV4 and GPR35 in young ECs, which was notably enhanced in aging ECs. Collectively, these findings suggest the presence of GPR35‐TRPV4 interaction in ECs and imply an increased interaction during aging. However, the functional implications of the interaction between TRPV4 and GPR35 remain to be fully elucidated.

### 3.3 TRPV4‐GPR35 Interaction Modulates EC Functions

**FIGURE 3:**
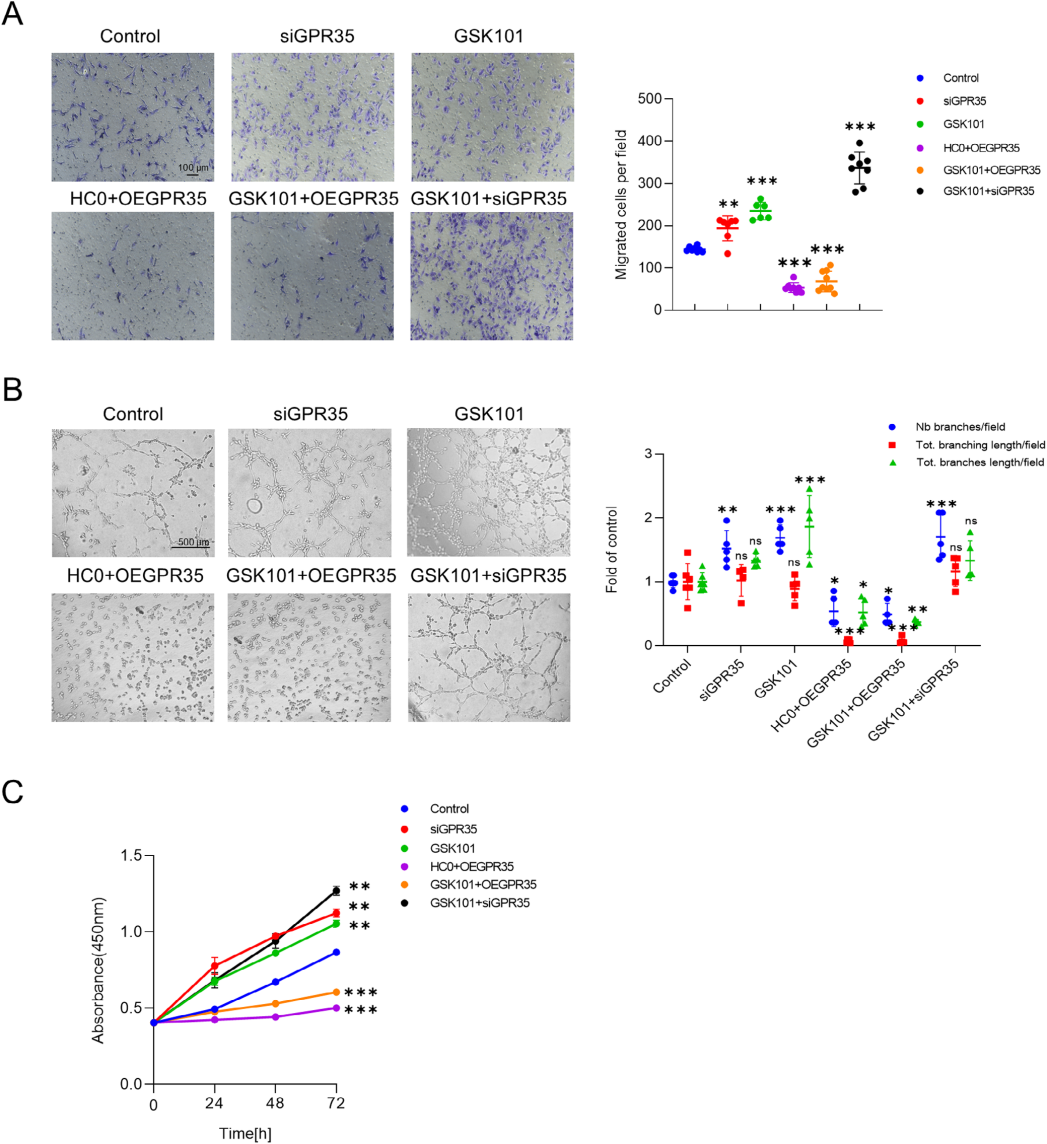
Regulation of EC functions by GPR35‐TRPV4 interaction. (A) Representative images showing migrated HUVECs in the cell migration assay: Control, normal HUVECs; siGPR35, HUVECs transfected with siGPR35 to knockdown GPR35 expression before experiments; OEGPR35, HUVECs transfected with GPR35 plasmid to overexpress GPR35 before experiments; GSK101, HUVECs treated with GSK101 (10 nM) during experiments; HC0, HUVECs treated with HC0 (5 μM) during experiments. Scale bar: 100 μm. Quantification of migrated cells per field between these groups is shown on the right (*n* = 5 for each group; **p < 0.01 vs. controls, ***p < 0.001 vs. controls; one‐way ANOVA). (B) HUVEC tube formation ability assessed under the same conditions as listed in (A). Scale bar: 500 μm. Comparisons of different groups are shown on the right (*n* = 4–7 for each group; *p < 0.05 vs. controls, **p < 0.01 vs. controls, ***p < 0.001 vs. control; ns indicates no statistical significance when comparing with controls; one‐way ANOVA). (C) Cell viability of HUVECs assessed by CCK‐8. Different treatments were the same as listed in (A), *n* = 3 for each group; **p < 0.01 vs. controls; ***p < 0.001 vs. controls; two‐way ANOVA.

GPR35 has previously been associated with EC functions in the literature (Li et al. 2021). To investigate whether TRPV4 modulates GPR35 to regulate endothelial functions during aging, a series of functional assays, including migration, tube formation, and CCK8 experiments, were conducted using HUVECs (Figure 3A–C). Cells were treated with a TRPV4 inhibitor (HC0) and overexpressed with GPR35 (OEGPR35) to simulate aging conditions. As anticipated, inhibition of TRPV4 function coupled with elevated GPR35 expression resulted in compromised EC functions. Conversely, inhibition of GPR35 or activation of TRPV4 significantly bolstered the migration, tube formation, and proliferation of HUVECs. Interestingly, all assay outcomes indicated that the functions of HUVECs were more dependent on the expression of GPR35 rather than TRPV4, when both proteins were manipulated simultaneously. These findings collectively suggest that TRPV4 may regulate GPR35 to mediate EC functions.

### 3.4 GPR35‐TRPV4 Interaction Facilitates Endothelial Ca^2+^ Signaling and Vasodilation

**FIGURE 4:**
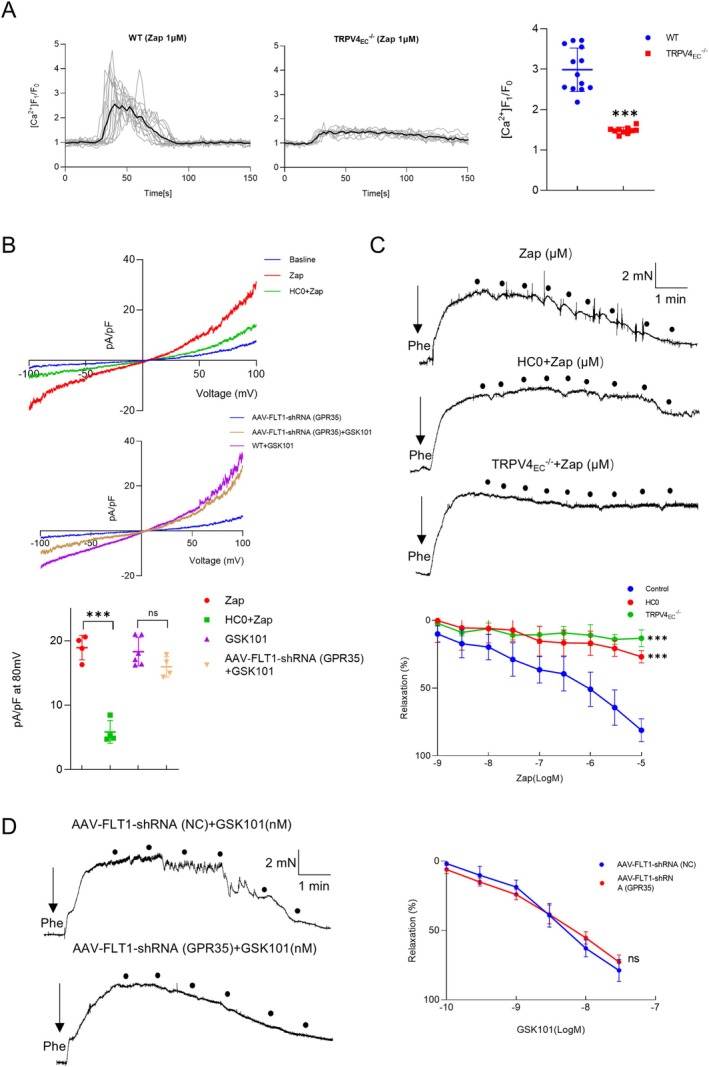
Modulation of endothelial Ca^2+^ signaling and vasodilation by GPR35‐TRPV4 interaction. (A) Left panel: Representative traces showing the time course of zaprinast (Zap, 1 μM)‐induced Ca^2+^ influx in primary mesenteric ECs from WT and TRPV4_EC_^−/−^ mice. Right panel: Summarized data of Ca^2+^ influx represented in (A) (*n* = 10–13 cells from 3 to 4 isolations for each group; ***p < 0.001 vs. WT ECs; *t*‐test). (B) Representative traces of TRPV4 channel currents from whole‐cell patch clamp recordings of isolated ECs from WT mice (top) challenged with Zap (1 μM) or Zap (1 μM) + HC0 (10 μM), and AAV‐FLT1‐shRNA (GPR35) mice (middle) with or without GSK101 (30 nM). Comparisons of the TRPV4 channel current densities at 80 mV between groups as listed above (bottom), *n* = 4–6 cells from 3 isolations for each group; ***p < 0.001, ns indicates no statistical significance, as indicated in the figure; *t*‐test. (C) Representative traces and summarized data depicting gradient concentrations of Zap‐induced vasodilation in mesenteric arteries from WT mice (top), in the presence of HC0 (10 μM) (middle), and TRPV4_EC_^−/−^ mice (bottom), *n* = 6–8 arteries for each group; ***p < 0.001 vs. WT controls; two‐way ANOVA. (D) Representative traces and summarized data depicting gradient concentrations of GSK101‐induced vasodilation in mesenteric arteries from AAV‐FLT1‐shRNA (NC) (top) and AAV‐FLT1‐shRNA (GPR35) (bottom) mice, *n* = 5–6 arteries for each group, ns indicates no statistical significance vs. AAV‐FLT1‐shRNA (NC) mice; two‐way ANNOVA.

TRPV4 channels are pivotal in endothelial Ca^2+^ signaling and vascular function (Chen and Sonkusare 2020; Sonkusare et al. 2012). Given the observed interaction between TRPV4 and GPR35 in primary ECs, it is crucial to determine whether GPR35 can modulate functional events through TRPV4. We discovered that zaprinast (Zap), a selective GPR35 agonist, induced Ca^2+^ influx in single primary ECs from WT mesenteric arteries (Figure 4A). Interestingly, Zap‐induced Ca^2+^ influx was significantly reduced in single ECs from TRPV4_EC_^−/−^ mesenteric arteries. To further confirm the role of TRPV4 in mediating Ca^2+^ currents induced by Zap, whole‐cell patch clamp recordings were conducted on freshly isolated ECs from WT third‐order mesenteric arteries. Zap markedly increased TRPV4 ionic currents compared with controls. However, this effect was abolished in the presence of HC0 (Figure 4B). Since Zap has been reported not only to activate GPR35 but also to act as a cGMP phosphodiesterase inhibitor, another selective GPR35 activator, kynurenic acid (KYNA), was also used. Similarly, KYNA induced endothelial Ca^2+^ influx and caused dose‐dependent vasodilation from WT mice (Figure S5). However, knocking out endothelial TRPV4 significantly attenuated these effects.

To gain deeper insights into the role of GPR35 in vivo, AAV‐FLT1‐shRNA (GPR35) mice were generated due to the unavailability of selective inhibitors. After confirmation (Figure S6), primary ECs from these mice were collected. Notably, GSK101 was still able to increase TRPV4 ionic currents in these cells (Figure 4B). Next, to assess whether GPR35‐TRPV4 interaction could induce vessel dilation, the tensions of small mesenteric arteries were measured using wire myography. Zap initiated a dose‐dependent relaxation of small mesenteric arteries. However, in the presence of HC0, Zap failed to induce vasodilation. Additionally, similar results were observed in TRPV4_EC_^−/−^ arteries (Figure 4C). Interestingly, when GPR35 was knocked down in the endothelium, GSK101 was still capable of inducing vasodilation (Figure 4D). Together, these findings indicate that GPR35 may function as an upstream regulator in TRPV4‐mediated Ca^2+^ signaling in ECs and vasodilation.

### 3.5 Small Molecule Compounds Restore Aging‐Induced Endothelial Dysfunction by Targeting GPR35‐TRPV4 Interaction

**FIGURE 5:**
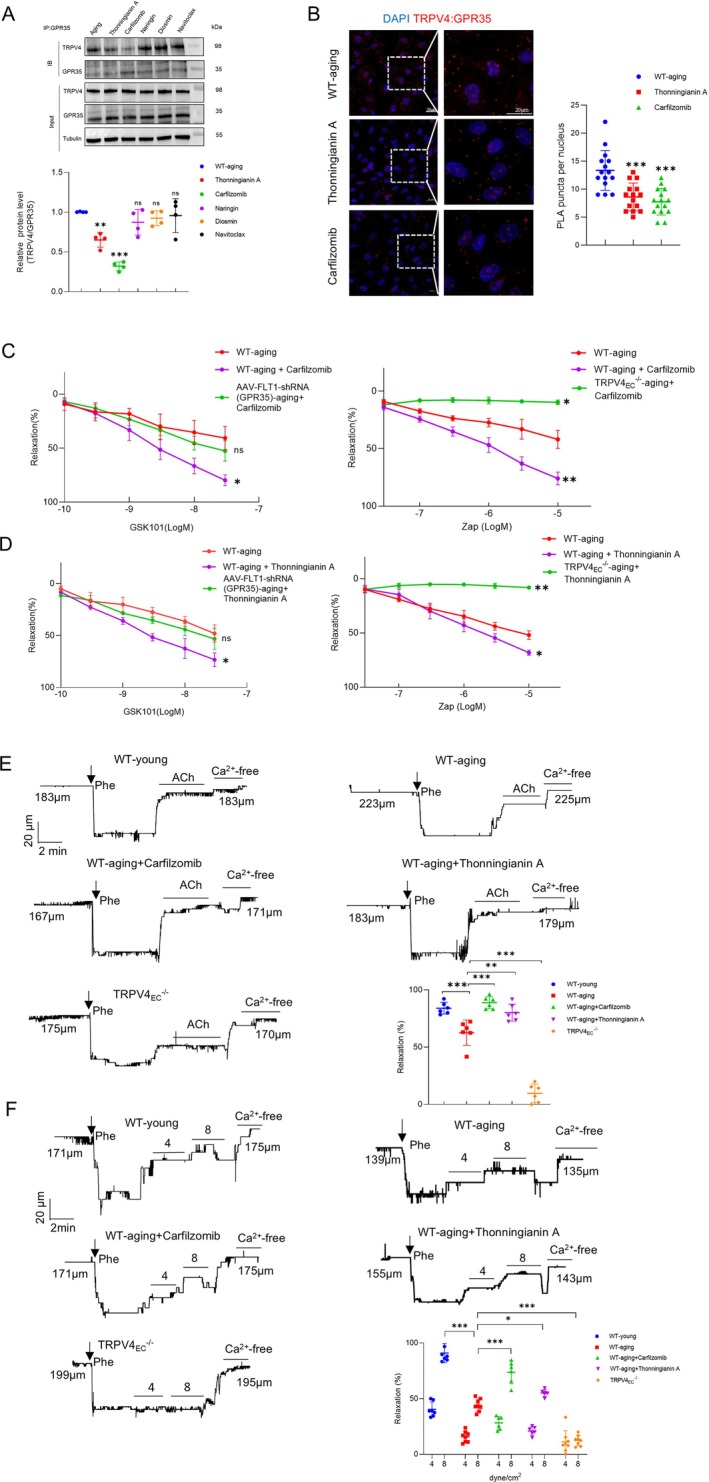
Small molecule compounds prevent impaired vasodilation by targeting GPR35‐TRPV4 interaction. (A) Top panel: Co‐immunoprecipitation (co‐IP) assay of TRPV4 and GPR35 in primary mesenteric ECs from aging mice incubated with Thonningianin A (10 μM), Carfilzomib (1 μM), Naringin (10 μM), Diosmin (10 μM), and Navitoclax (1 μM) for 30 min before harvest. The lysates were immunoprecipitated with control IgG or GPR35 antibody, followed by immunoblotting (IB) with TRPV4 antibody. The quantitative analysis is shown on the bottom, *n* = 4 isolations for each group; ***p < 0.001 vs. aging ECs, ns indicates no statistical significance vs. aging ECs, one‐way ANOVA. (B) Left Panel: Representative merged images of proximity ligation assays (PLAs) signal, showing EC nuclei and TRPV4_EC_‐GPR35_EC_ co‐localization (red puncta) in primary ECs from third‐order mesenteric arteries from WT‐aging mice treated with Thonningianin A (10 μM) and Carfilzomib (1 μM). Scale bar: 20 μm. Right panel: Quantification of TRPV4_EC_‐GPR35_EC_ co‐localization (*n* = 15 cells from 4 to 5 isolations for each group; ***p < 0.001 vs. WT‐aging group; one‐way ANOVA). (C) The dose‐dependent of GSK101 (left) and Zap (right)‐induced vasorelaxation of mesenteric arteries from the groups of WT‐aging, AAV‐FLT1‐shRNA (GPR35)‐aging and TRPV4_EC_^−/−^ aging treated with Carfilzomib (*n* = 3–4 arteries for each group; *p < 0.05, **p < 0.01 and ns indicates no statistical significance vs. WT‐aging mice; two‐way ANOVA). (D) The dose‐dependent of GSK101 (left) and Zap (right)‐induced vasorelaxation of mesenteric arteries from the groups of WT‐aging, AAV‐FLT1‐shRNA (GPR35)‐aging and TRPV4_EC_^−/−^ aging treated with Thonningianin A (*n* = 3–4 arteries for each group; *p < 0.05, **p < 0.01 and ns indicates no statistical vs. WT‐aging mice; two‐way ANOVA). (E) Representative traces of 10 μM acetylcholine (ACh)‐induced vasodilation of third‐order mesenteric arteries from WT‐young (top left), WT‐aging (top right), WT‐aging + Carfilzomib/Thonningianin A (middle), and TRPV4_EC_^−/−^ (bottom left) groups. Bottom right: Comparisons of the percentages of dilation induced by ACh between the indicated groups, *n* = 6 arteries for each group; **p < 0.01, ***p < 0.001 vs. WT aging arteries or young arteries as indicated in the figure; one‐way ANOVA. (F) Representative traces of 4/8 dyne/cm^2^ flow‐induced vasodilation of third‐order mesenteric arteries from WT‐young (top left), WT‐aging (top right), WT‐aging + Carfilzomib/Thonningianin A (middle), and TRPV4_EC_^−/−^ (bottom left) groups. Bottom right: Comparisons of the percentages of dilation induced by flow between the indicated groups, *n* = 6–8 arteries for each group; ***p < 0.001 vs. WT aging arteries or young arteries as indicated in the figure; one‐way ANOVA.

Given the pivotal role of GPR35‐TRPV4 interaction in regulating endothelial functions, it is imperative to explore precise therapeutic strategies to address aging‐related endothelial dysfunction. TRPV4, as one of the most important Ca^2+^ permeable cation channels, is widely distributed and exerts vital functions in various tissues. However, systemic blockade of TRPV4 may lead to widespread side effects. Hence, it is essential to devise novel approaches that specifically target key components, such as the GPR35‐TRPV4 interaction, to mitigate unwanted effects. Based on the dominant binding sites, we screened a series of natural compounds with the strongest affinity to decrease GPR35‐TRPV4 interaction using LibDock (Table S3). Co‐IP assays were then conducted to validate whether the candidate compounds could inhibit the GPR35‐TRPV4 interaction using primary ECs from WT‐aging mesenteric arteries. Results indicated that Thonningianin A and Carfilzomib effectively decreased the GPR35‐TRPV4 interaction, whereas naringin, diosmin, and navitoclax did not (Figure 5A). Subsequent PLA assays confirmed that Thonningianin A and Carfilzomib significantly reduced the co‐localization of TRPV4 and GPR35, as shown in Figure 5B. We then used receptor–ligand interactions tool to predict the binding potential of Thonningianin A and Carfilzomib to these proteins. As illustrated in Figure S7, Thonningianin A is predicted to bind to TRPV4 at ARG249 (adjacent to ARG248, as listed in Table S2), while Carfilzomib is predicted to bind to TRPV4 at ASN296 (adjacent to ASN338) and GPR35 at ARG280 (same as ARG280 in Table S2). Notably, the same concentrations of Thonningianin A and Carfilzomib did not significantly decrease the GPR35‐TRPV4 interaction in WT‐young primary ECs (Figure S8). Further analysis evaluated the effects of Carfilzomib on vasodilation in mesenteric arteries from aging mice (Figure 5C). Carfilzomib treatment significantly enhanced the maximal vasorelaxation response to GSK101 in aging arteries compared to untreated controls. However, knockdown of endothelial GPR35 did not reverse the vasodilatory effects of Carfilzomib in aging arteries. Additionally, while Carfilzomib substantially increased Zap‐induced maximal vasodilation in WT‐aging arteries, this effect was abolished in TRPV4_EC_^−/−^ aging arteries. Similarly, Thonningianin A markedly improved vasodilation in aging arteries but lost efficacy in GPR35‐knockdown and TRPV4_EC_^−/−^ mice (Figure 5D). Collectively, these results suggest that the observed improvements in arterial relaxation are mediated by restoring of the GPR35‐TRPV4 interaction, rather than through direct targeting of TRPV4 or GPR35, when treated with Carfilzomib or Thonningianin A.

To further elucidate the functional impact of Thonningianin A and Carfilzomib, we initially examined their effects on EC function in aging. Intriguingly, both Thonningianin A and Carfilzomib exhibited the ability to ameliorate impaired cell functions in primary aging ECs (Figure S9). Subsequently, we evaluated their effects on vasodilation induced by endogenous vessel relaxants to simulate physiological conditions. This included treatment with 10 μM acetylcholine (ACh) and shear stress at 4 or 8 dyne/cm^2^, and we measured the diameters of mesenteric arteries using a pressure myograph. A concentration of 10 μM ACh is sufficient to induce maximal dilation of mesenteric arteries, as demonstrated in previous studies (He et al. 2017; Zhang et al. 2009). As anticipated, aging arteries exhibited impaired dilation compared to young arteries under ACh and shear stress. However, both Thonningianin A and Carfilzomib were able to restore vasodilation impairment in aging arteries under these conditions (Figure 5C,D). Remarkably, Carfilzomib elicited greater arterial dilation compared to Thonningianin A, particularly under flow conditions. Collectively, these findings suggest that restoring of the GPR35‐TRPV4 association by Thonningianin A or Carfilzomib could effectively prevent aging‐related endothelial dysfunction and vasodilation impairment.

## 4 Discussion

Endothelial dysfunction, including impaired vasodilation, plays a critical role in the pathogenesis of various aging‐related cardiovascular diseases (Donato, Machin, and Lesniewski 2018; Herrera et al. 2010). Despite the established importance of TRPV4 channels in regulating vascular reactivity in previous literature, the specific role of TRPV4 and the underlying mechanisms in aging‐related endothelial dysfunction remain incompletely understood. In our study, we found that the loss of TRPV4 function, rather than its expression, significantly contributes to aging‐related vasodilation impairment. Mechanistically, we identified that GPR35‐TRPV4 interaction mediates endothelial function and vascular tone. In aging small resistance arteries, endothelial GPR35 expression is significantly elevated, leading to abnormally enhanced GPR35‐TRPV4 interaction, which is correlated with impaired vasodilation. Furthermore, compounds such as Thonningianin A and Carfilzomib effectively protect against endothelial dysfunction and impaired vasodilation by restoring the GPR35‐TRPV4 interaction, suggesting a new precise therapeutic strategy for preventing vasodilation impairment in aging arteries.

The regulation of vascular TRPV4 channels appears to exhibit variability across species, different vascular beds, and various animal models (Goto and Kitazono 2021). In studies involving rats, diminished TRPV4 expression and function have been associated with endothelial dysfunction in mesenteric arteries under conditions of genetic and salt‐induced hypertension (Boudaka et al. 2019; Gao et al. 2009; Seki et al. 2017), obesity (Ma et al. 2013), and aging (Du et al. 2016). Conversely, in aortic arteries of salt‐induced hypertensive rats, TRPV4 expression remained largely unchanged, yet an increase in TRPV4‐induced constriction was observed (Zhang et al. 2018). In diabetic rats, reduced TRPV4 expression in aortas led to impaired vasorelaxation (Shamsaldeen, Lione, and Benham 2020). Notably, in mouse models of angiotensin II‐induced and salt‐induced hypertension, consistent reports indicate reduced TRPV4 function without alterations in expression across multiple studies (He et al. 2017; Nishijima et al. 2014; Sonkusare et al. 2014). In line with existing evidence, our study highlights the close relationship between impaired vasodilation and diminished TRPV4 function without changes in expression in the third‐order mesenteric artery of aging mice (Figure 1, Figure S3). Cumulatively, both our findings and those of other research teams underscore the significant contribution of reduced TRPV4 function, independent of alterations in expression, to impaired vasodilation in small resistant arteries across various pathological models. However, further investigations are warranted to comprehensively elucidate the changes in TRPV4 function and expression during different pathological processes.

The primary discovery of our study is the identification of the regulatory role of the GPR35‐TRPV4 interaction in endothelial function. To our knowledge, this study marks the first time that an enhanced GPR35‐TRPV4 interaction has been linked to significant contributions to endothelial dysfunction and impaired vasodilation in small resistance arteries of aging mice. GPR35, a recently deorphanized GPCR, has garnered attention as a potential therapeutic target due to its associations with various pathologies, including heart failure (Divorty et al. 2018; Ronkainen et al. 2014), diabetes (Horikawa et al. 2000) and inflammatory diseases (Farooq et al. 2018). However, GPR35 has not been extensively linked to endothelial dysfunction, except for a single study demonstrating its involvement in endothelial function and vascular tone modulation (Li et al. 2021). In this study, researchers found that GPR35 deletion offers protection of EC function during deoxycorticosterone acetate‐salt‐induced hypertension. Consistently, we observed significantly elevated expression of GPR35 in aging primary ECs compared to young controls (Figure 2B,C). Importantly, we also uncovered that TRPV4 can act as an upstream regulatory protein to GPR35 in mediating EC functions (Figure 3). Additionally, both endothelial TRPV4 and GPR35 have been reported to modulate vascular tone (Li et al. 2021; Sonkusare et al. 2012). Our study indicates that GPR35 may contribute to vascular tone modulation through TRPV4 (Figure 4). Given the close relationship between TRPV4 and GPR35, our study provides supportive evidence that the interaction between these two proteins plays a crucial role in endothelial function and vascular tone modulation.

Another notable aspect of the current study is the discovery of Thonningianin A and Carfilzomib as potential therapeutic agents for mitigating aging‐related endothelial dysfunction impaired vasodilation by restoring the interaction between TRPV4 and GPR35. Considering that TRPV4 is widely distributed and plays a predominant role in various tissues, there is a concern that systemic blockade of TRPV4 could lead to undesirable adverse effects. In light of this, our research group has shifted focus towards targeting protein complexes to modulate specific signaling pathways, aiming for more precise interventions with fewer side effects. For example, previous studies have shown that improving impaired endothelial TRPV4‐KCa2.3 coupling can protect against the progression of hypertension (He et al. 2017). Similarly, small molecules have been developed to restore decreased TRPV4–eNOS interaction and treat hypertension (Mao et al. 2022). Furthermore, repairing the uncoupled TRPV4‐KCa3.1 channels with folic acid has been shown to effectively enhance coronary arterial function in mice (Mao et al. 2023). Building on these insights, we predicted potential binding sites between TRPV4 and GPR35 and screened a series of compounds using molecular docking to identify those with the strongest affinity for decreasing GPR35‐TRPV4 interaction (Figure 5A,B). Subsequent assays confirmed that Thonningianin A and Carfilzomib effectively prevented abnormal EC functions and vasorelaxation impairment by restoring the GPR35‐TRPV4 association, without affecting TRPV4 expression (Figure 5C,D). This discovery offers a novel strategy for the specific treatment of aging‐related vasodilation impairment.

Lastly, our study has several limitations. Previous research has shown that the loss of endothelial TRPV4 promotes lung cancer, and TRPV4 activation inhibits proliferation (Kanugula et al. 2021; Thoppil et al. 2015). However, our data suggests that TRPV4 activation significantly increases the migration, tube formation, and proliferation of HUVECs. These differing effects may be specific to certain vascular beds, warranting further investigation. Additionally, we observed that increased GPR35 expression in aging enhances the interaction between GPR35 and TRPV4, which inhibits TRPV4‐associated vasodilation. We propose that this heightened interaction may drive TRPV4 translocation in the membrane, impairing its function, as certain TRP channels translocate to the membrane upon cellular stimulation. Further research is needed to explore the location and association of TRPV4 and GRP35.

## 5 Conclusions

In summary, our study revealed the significant involvement of GPR35‐TRPV4 interaction in endothelial function and the modulation of vascular tone. We observed that abnormally enhanced GPR35‐TRPV4 interaction contributed to impaired vasodilation of mesenteric arteries in aging mice. Restoring GPR35‐TRPV4 interaction with Thonningianin A/Carfilzomib effectively prevented the progression of vasodilation impairment in aging models. These findings offer valuable insights into potential preventive and therapeutic strategies for addressing aging‐related endothelial dysfunction.

## Author Contributions

L.G., X.T., H.K., L.Y., Z.W. and T.Z. performed the experiments and analyzed the data. L.G., and X.T. wrote the manuscript. K.Z., A.M. and X.W. performed the RNA‐seq analysis. T.Z., X.W., X.Z. and L.F. discussed the study and edit the manuscript. L.G. designed the study and provided overall supervision.

## Conflicts of Interest

The authors declare no conflicts of interest.

## Supporting information


Appendix S1

## Data Availability

The data that support the findings of this study are available from the corresponding author upon reasonable request.
