# Supplementary material for: Investigating the Role of TRPV4 and GPR35 Interaction in Endothelial Dysfunction in Aging Mice
Source: Aging Cell. 2025 Jan 2;24(5):e14469. doi: 10.1111/acel.14469 (PMC12074021; doi:10.1111/acel.14469)
Supplement: Supplementary file 1 — Appendix S1 [file ACEL-24-e14469-s001.docx]

**fSupplemental Materials for**

**Investigating the Pivotal Role of TRPV4 and GPR35 Interaction in Endothelial Dysfunction in Aging Mice**

| Phlda1  Young vs. aging, p=0.00136;  WT vs. KO, p=0.00071 | Pleckstrin Homology Like Domain Family A Member 1 | anti-apoptotic and pro-apoptosis in metabolic diseases^1,2^, involve in the development of multiple of cancers^3^ |
| --- | --- | --- |
| Fosl1  Young vs. aging, p<0.00001; WT vs. KO, p=0.02757 | FOS Like 1, AP-1 Transcription Factor Subunit | Wnt-ß-Catenin-FOSL signaling can ameliorates right ventricular remodeling^4^ |
| GPR35  Young vs. aging, p=0.00397; WT vs. KO, p=0.01691 | G Protein-Coupled Receptor 35 | regulate intestinal homeostasis^5,6^, endothelial cell proliferation^7^, affecting blood pressure regulation^8^ |
| Rnd1  Young vs. aging, p=0.00096; WT vs. KO, p=0.00386 | Rho family GTPase 1 | inhibit epithelial-mesenchymal transition in hepatocellular carcinoma^9^ |
| Mmp13  Young vs. aging, p=0.00022; WT vs. KO, p=0.021027 | Matrix Metallopeptidase 13 | the protease responsible for collagen degradation in cartilage during osteoarthritis^10^ |
| Bdkrb2  Young vs. aging, p=0.00017; WT vs. KO, p= 0.021830 | Bradykinin B2 Receptor | mediate the majority of bradykinin effects, including vasodilation, release of nitric oxide and promotes water and sodium excretion^11,12^ |

Table S1: Candidate genes with elevated expression levels in both aging and TRPV4_EC_^-/-^ groups compared to WT controls (KO, TRPV4_EC_^-/-^).

1 Toyoshima Y, Karas M, Yakar S, Dupont J, Lee H, LeRoith D. TDAG51 mediates the effects of insulin-like growth factor I (IGF-I) on cell survival*.* *J Biol Chem* 2004;**279**:25898-904.

2 Park CG, Lee SY, Kandala G, Lee SY, Choi Y. A novel gene product that couples TCR signaling to Fas(CD95) expression in activation-induced cell death*.* *Immunity* 1996;**4**:583-91.

3 Zhao SB, Ma HJ, Wu ZG, Ling B, Ye GB. [Research Progress of Pleckstrin Homology Like Domain Family A Member 1 in Tumor]*.* *Zhongguo Yi Xue Ke Xue Yuan Xue Bao* 2022;**44**:863-7.

4 Nayakanti SR, Friedrich A, Sarode P, Jafari L, Maroli G, Boehm M, et al. Targeting Wnt-ß-Catenin-FOSL Signaling Ameliorates Right Ventricular Remodeling*.* *Circ Res* 2023;**132**:1468-85.

5 Kaya B, Doñas C, Wuggenig P, Diaz OE, Morales RA, Melhem H, et al. Lysophosphatidic Acid-Mediated GPR35 Signaling in CX3CR1(+) Macrophages Regulates Intestinal Homeostasis*.* *Cell Rep* 2020;**32**:107979.

6 Sun T, Xie R, He H, Xie Q, Zhao X, Kang G, et al. Kynurenic acid ameliorates NLRP3 inflammasome activation by blocking calcium mobilization via GPR35*.* *Front Immunol* 2022;**13**:1019365.

7 McCallum JE, Mackenzie AE, Divorty N, Clarke C, Delles C, Milligan G, et al. G-Protein-Coupled Receptor 35 Mediates Human Saphenous Vein Vascular Smooth Muscle Cell Migration and Endothelial Cell Proliferation*.* *J Vasc Res* 2015;**52**:383-95.

8 Li H, Nguyen H, Meda Venkata SP, Koh JY, Kowluru A, Li L, et al. Novel Role of GPR35 (G-Protein-Coupled Receptor 35) in the Regulation of Endothelial Cell Function and Blood Pressure*.* *Hypertension* 2021;**78**:816-30.

9 Qin CD, Ma DN, Zhang SZ, Zhang N, Ren ZG, Zhu XD, et al. The Rho GTPase Rnd1 inhibits epithelial-mesenchymal transition in hepatocellular carcinoma and is a favorable anti-metastasis target*.* *Cell Death Dis* 2018;**9**:486.

10 Li JJ, Johnson AR. Selective MMP13 inhibitors*.* *Med Res Rev* 2011;**31**:863-94.

11 Regoli D, Barabé J. Pharmacology of bradykinin and related kinins*.* *Pharmacol Rev* 1980;**32**:1-46.

12 Katori M, Majima M. Pivotal role of renal kallikrein-kinin system in the development of hypertension and approaches to new drugs based on this relationship*.* *Jpn J Pharmacol* 1996;**70**:95-128.

Potential binding sites of GPR35.

| Mutation | Mutation Energy | Effect of Mutation |
| --- | --- | --- |
| A:LYS287>ALA | 1.95 | DESTABILIZING |
| A:GLN283>ALA | 1.63 | DESTABILIZING |
| A:GLN212>ALA | 1.42 | DESTABILIZING |
| A:ARG280>ALA | 1.4 | DESTABILIZING |
| A:LYS213>ALA | 1.14 | DESTABILIZING |
| A:GLY206>ALA | 0.83 | DESTABILIZING |
| A:PRO295>ALA | 0.53 | DESTABILIZING |

Potential binding sites of TRPV4.

| Mutation | Mutation Energy | Effect of Mutation |
| --- | --- | --- |
| A:GLY270>ALA | 5.1 | DESTABILIZING |
| A:GLY280>ALA | 2.04 | DESTABILIZING |
| A:PHE272>ALA | 2.03 | DESTABILIZING |
| A:GLU337>ALA | 1.68 | DESTABILIZING |
| A:TYR281>ALA | 1.48 | DESTABILIZING |
| A:ARG248>ALA | 0.8 | DESTABILIZING |
| A:ASN338>ALA | 0.61 | DESTABILIZING |

Table S2. Potential binding sites between GPR35 and TRPV4 by molecular docking.


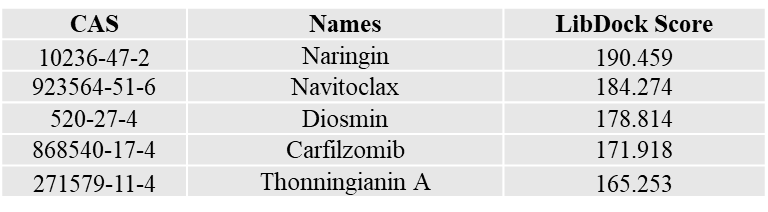
Table S3: Compounds with the strongest affinity to disrupt the TRPV4-GPR35 interaction using LibDock


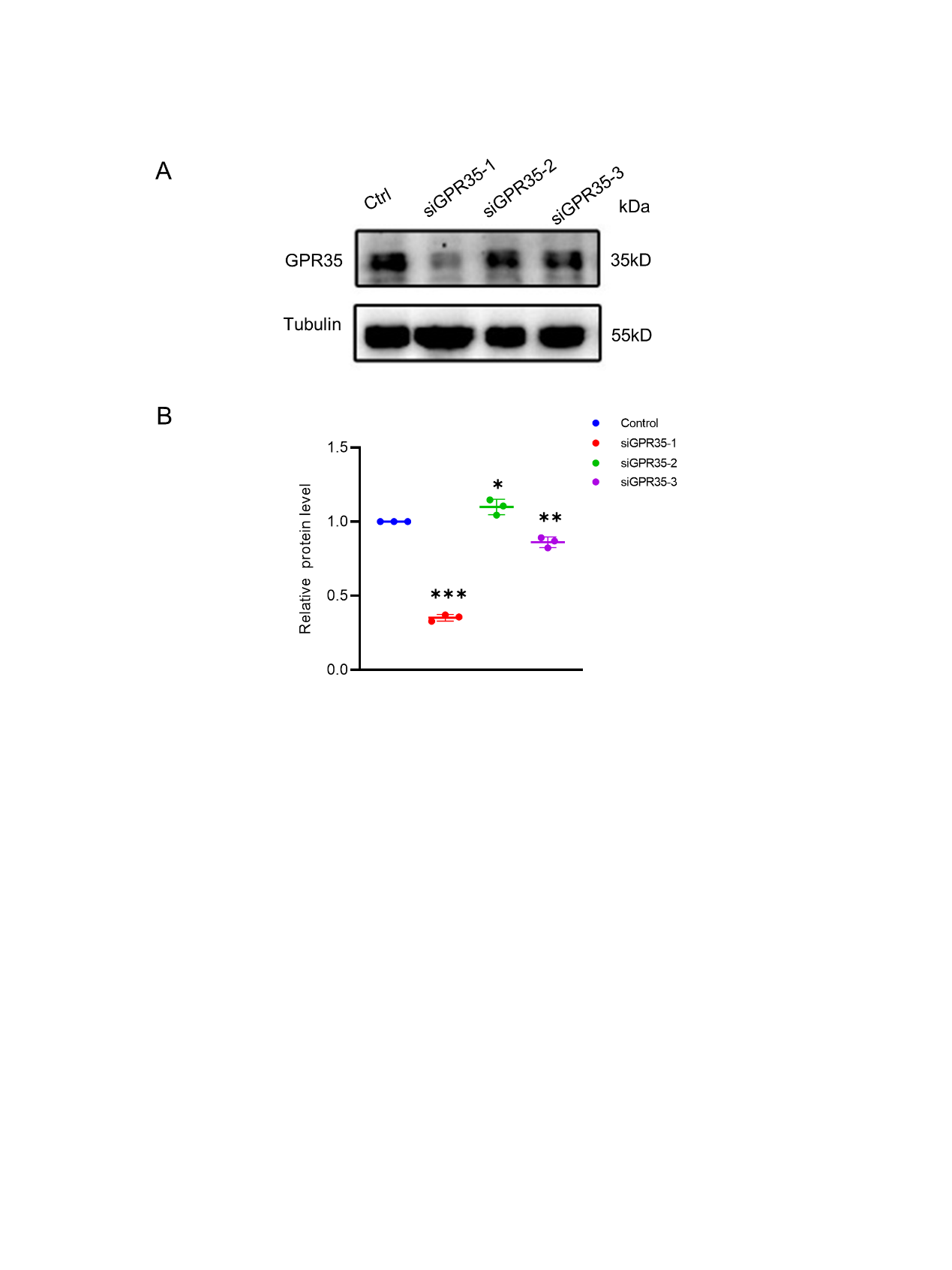
Figure S1: The efficiency of GPR35 siRNA in HUVECs. West blots (A) and qRT‒PCR analysis (B) of GPR35 protein and mRNA levels in HUVECs transfected with scramble siRNA (control) or three different siRNAs for 48 hours. The siRNA that demonstrated the most significant knockdown of GPR35 expression was selected for further experiments. n=3 for each group; *p<0.05, **p<0.01, ***p<0.001 vs. control group; one-way ANOVA.


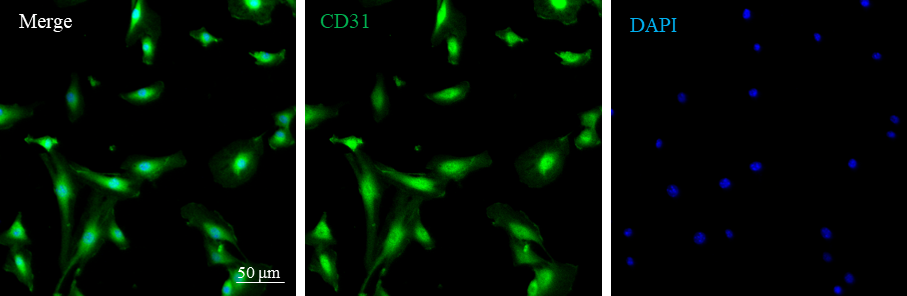
Figure S2: Immunofluorescence staining of CD31 in primary ECs isolated from mesenteric arteries. Representative images show CD31 (green) staining confirming endothelial identity and DAPI (blue) staining for nuclei.


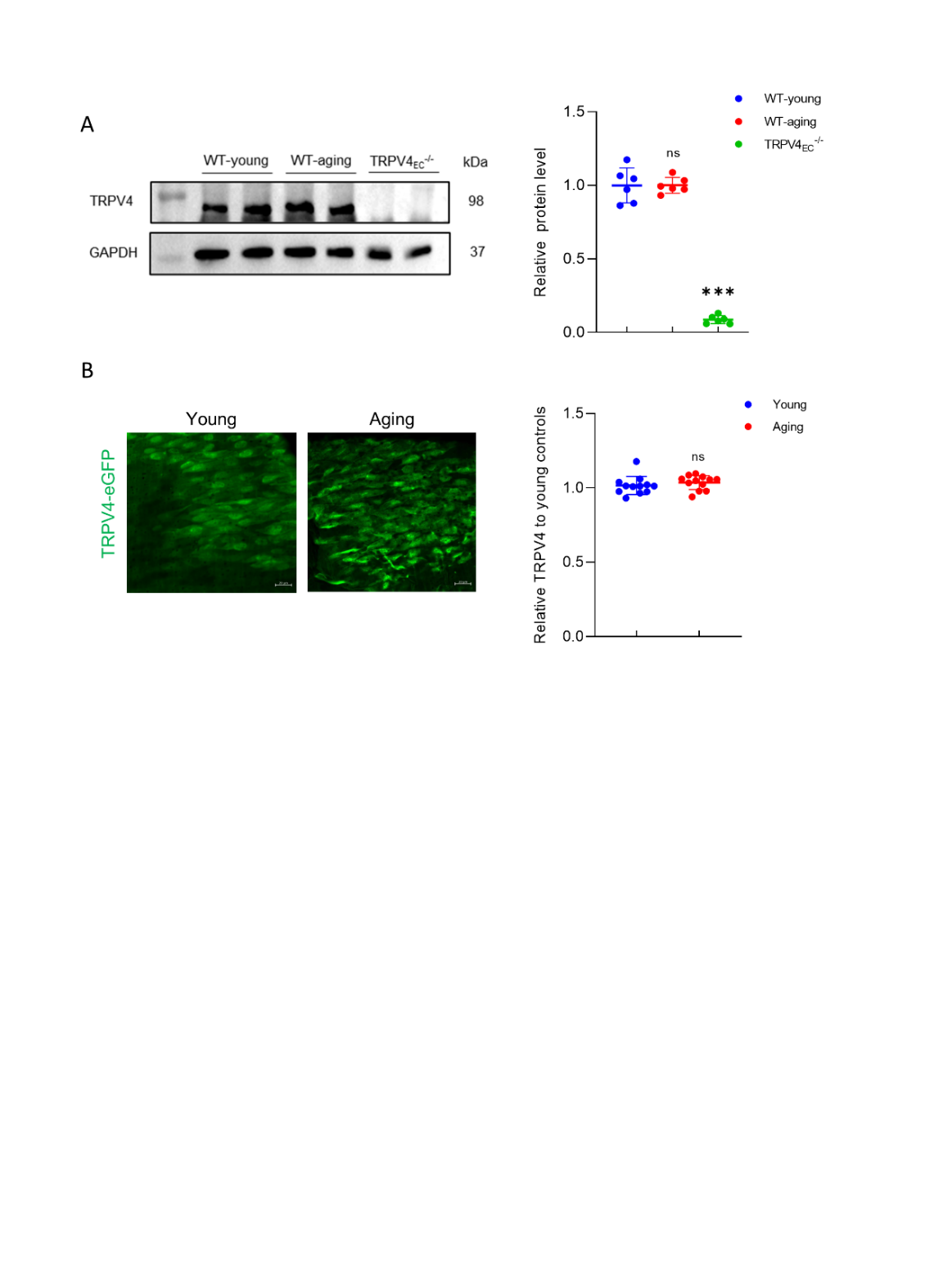
Figure S3: No significant differences in TRPV4 expression were found between WT young and aging mice. (A) Western blots for TRPV4 expression of primary mesenteric ECs from WT-young, WT-aging and TRPV4_EC_^-/-^ mice, n=6 for each group, ns indicates no statistical significance vs. WT-young ECs, ***p<0.001 vs. WT-young ECs, one-way ANOVA. (B) Third-order mesenteric arteries of TRPV4-eGFP mice were isolated and prepared en face before imaging. Scale bar: 20 μm. Comparisons were made between young and aging groups, n=12 fields from 3 mice for each group, ns indicates no statistical significance vs. young TRPV4-eGFP mice, t-test.

Figure S4: Volcano plot of differentially expressed genes, where significantly different genes are represented by red dots for upregulation and blue dots for downregulation. WY, WT-young; WA, WT-aging; KY, TRPV4_EC_^-/-^-young; KA, TRPV4_EC_^-/-^-aging.


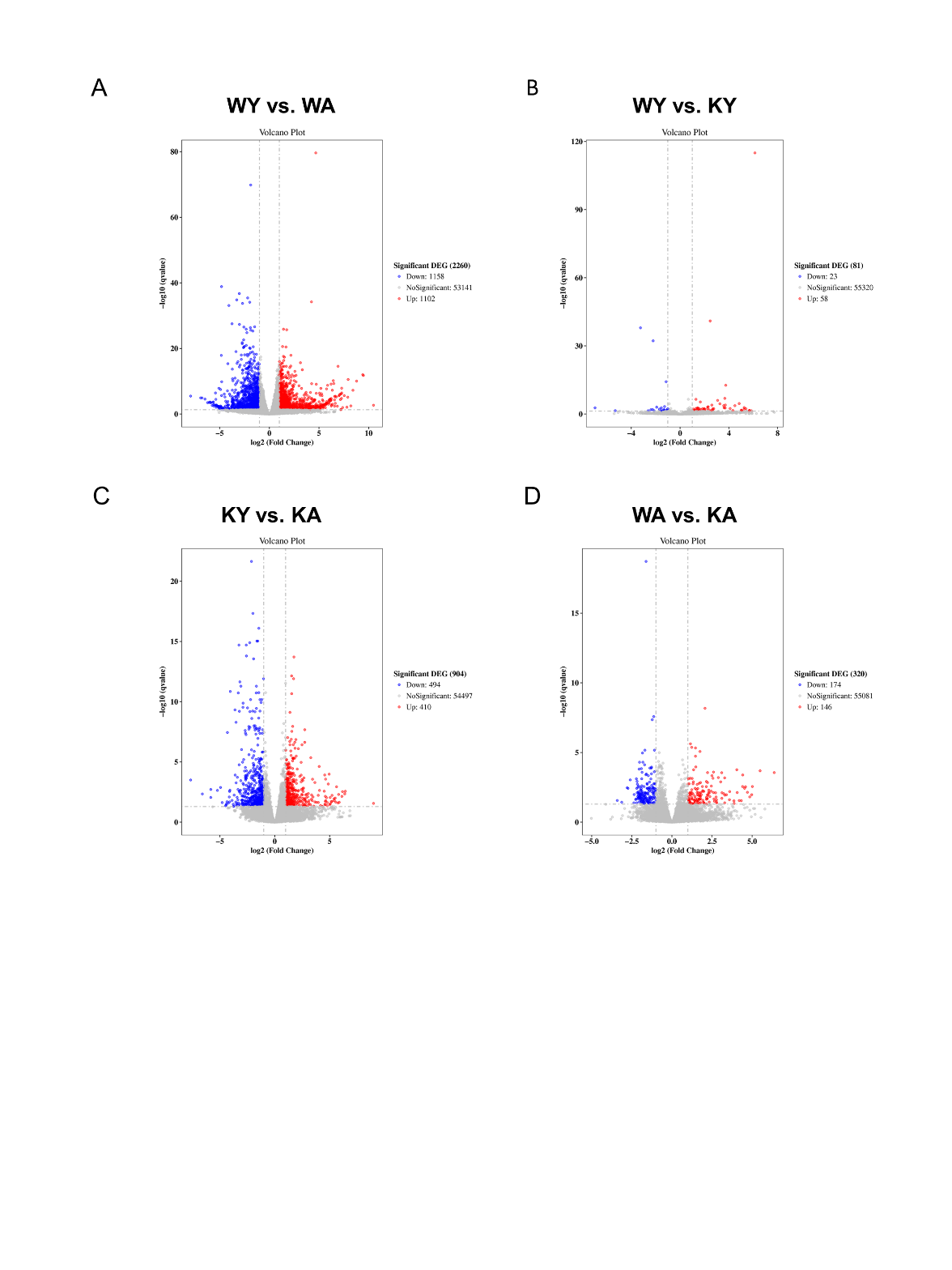


Figure S5. Modulation of endothelial Ca^2+^ signaling and vasodilation by KYNA. (A) Left panel: representative traces showing the time course of KYNA (10 μM)-induced
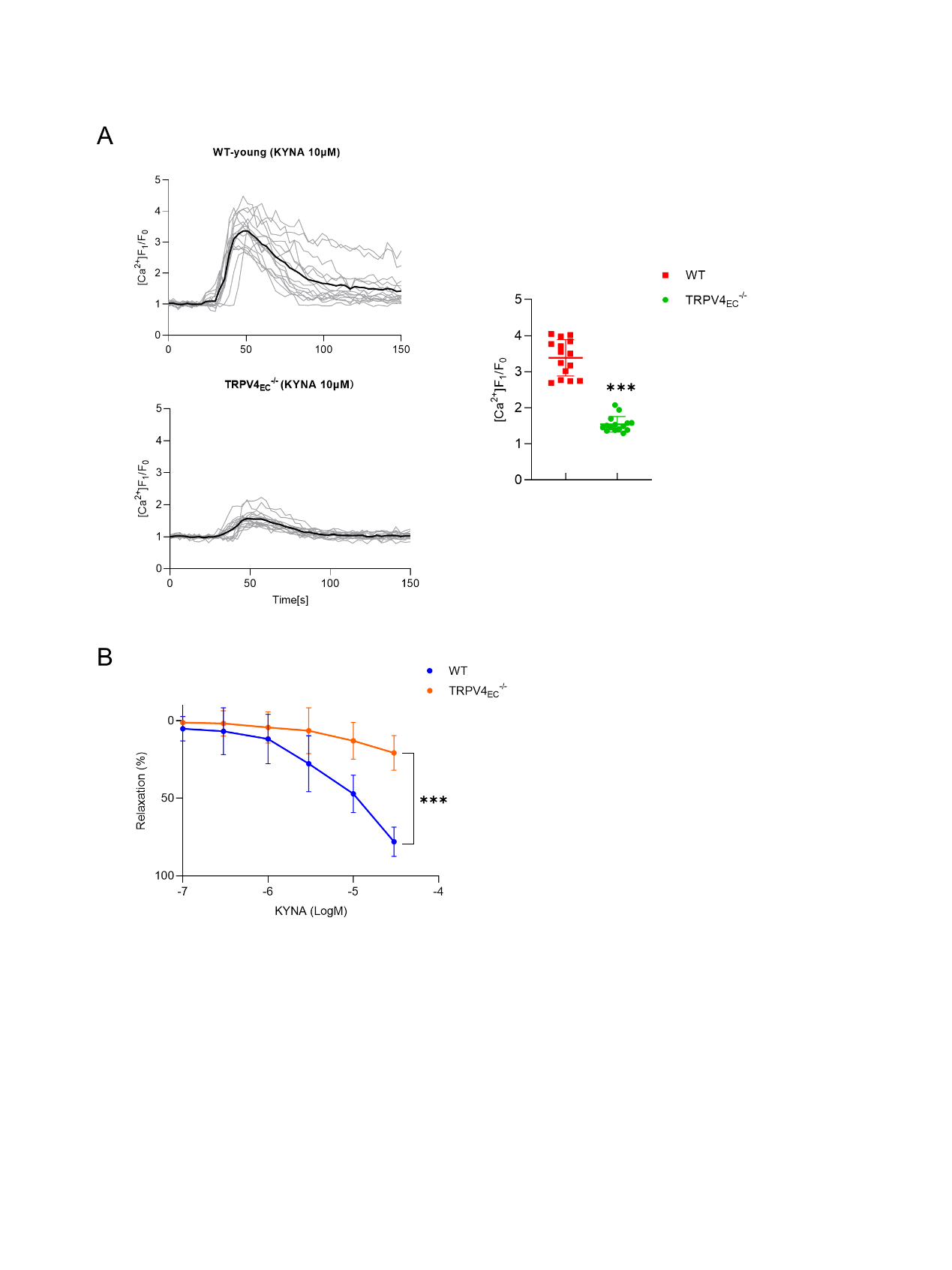
Ca^2+^ influx in primary mesenteric ECs from WT and TRPV4_EC_^-/-^ mice. Right panel: summarized data of Ca^2+^ influx represented in (A) (n=15 cells from 3-4 isolations for each group; ***p<0.001 vs. WT ECs; t-test). (B) Summarized data depicting gradient concentrations of KYNA-induced vasodilation in mesenteric arteries from WT and TRPV4_EC_^-/-^ mice, n=6 arteries for each group; ***p<0.001 vs. WT controls; two-way ANNOVA.

Figure S6: Confirmation of the generation of AAV-FLT1-shRNA (GPR35) mice. (A) Western blot analysis revealed significantly lower expression levels of GPR35 in isolated mesenteric ECs from AAV-FLT1-shRNA (GPR35) mice compared to AAV-FLT1-shRNA (NC) mice. (B) Third-order mesenteric arteries from AAV-FLT1-shRNA (GPR35) and AAV-FLT1-shRNA (NC) mice were isolated and prepared *en face* before imaging. Red fluorescence indicates successful transfection with AAV. Scale bar: 20 μm.


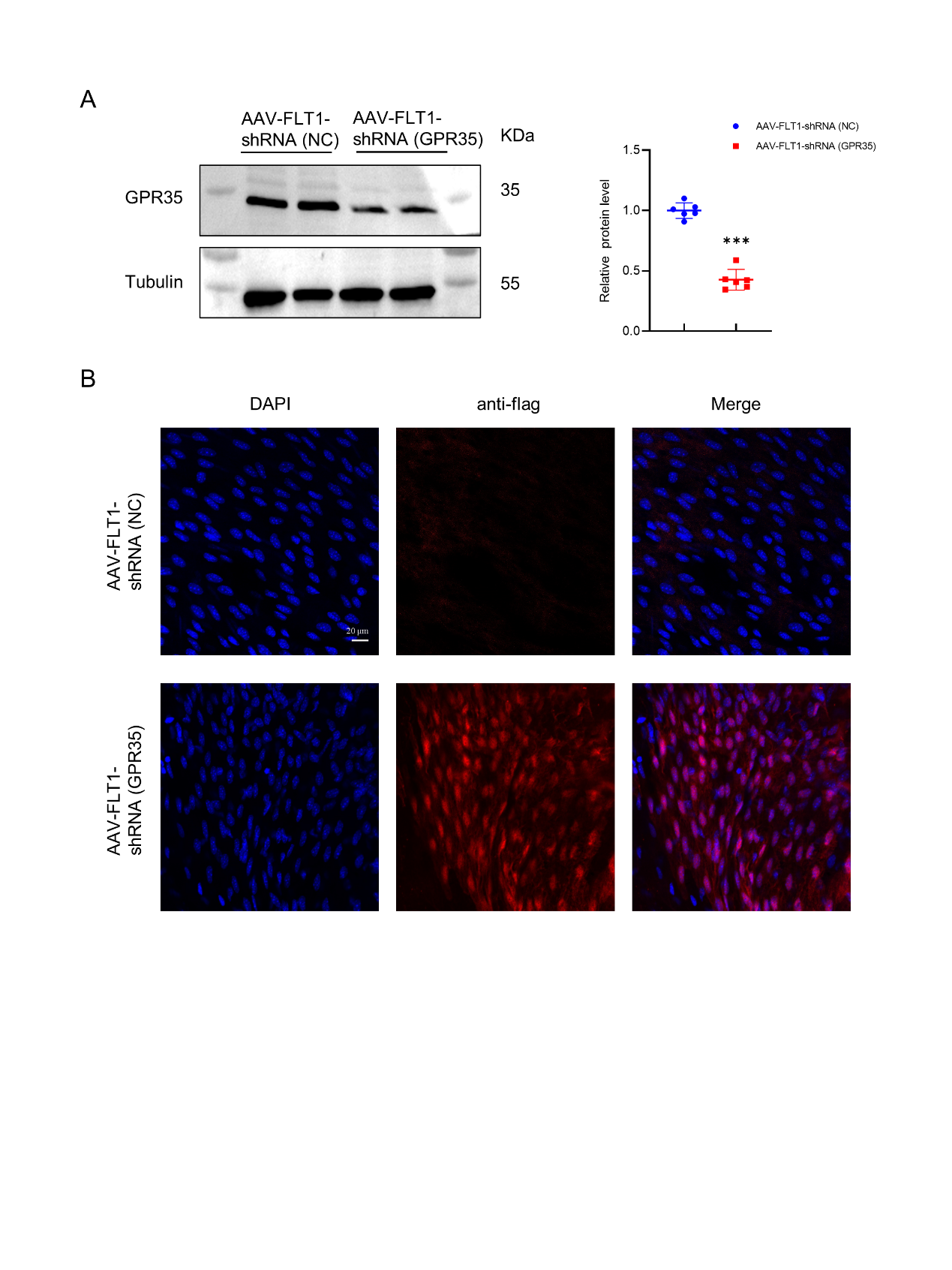


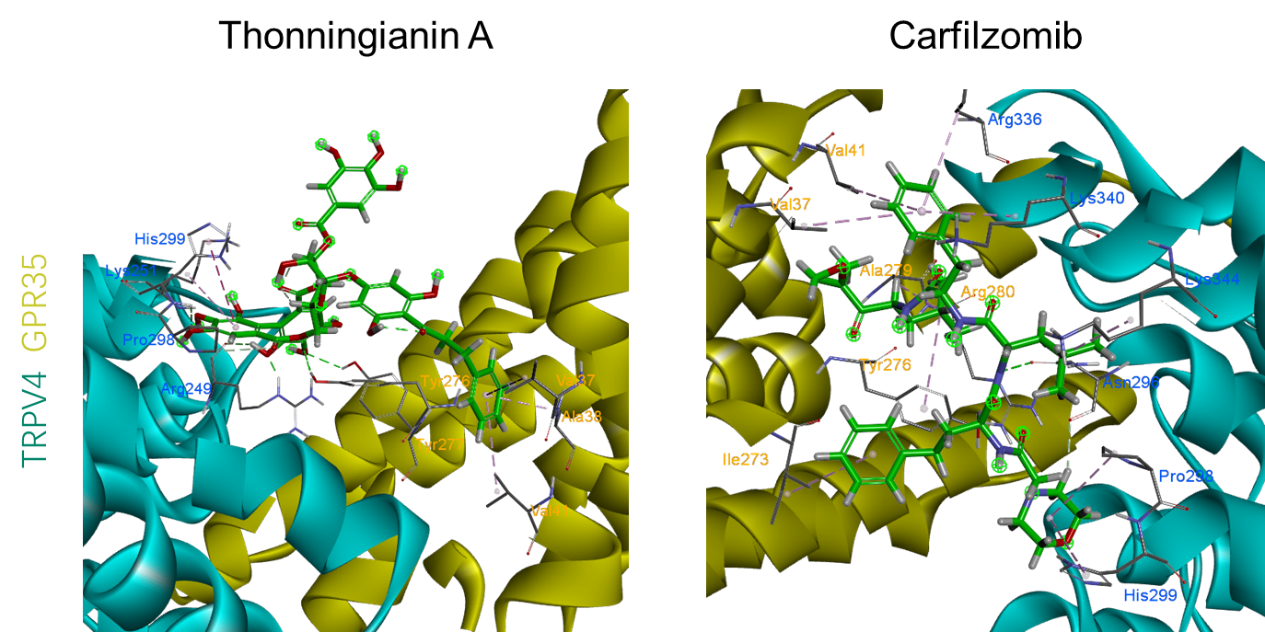


Figure S7: Molecular docking analysis showed the 3D structures of Thonningianin A (left) and Carfilzomib (right) between TRPV4 and GPR35.


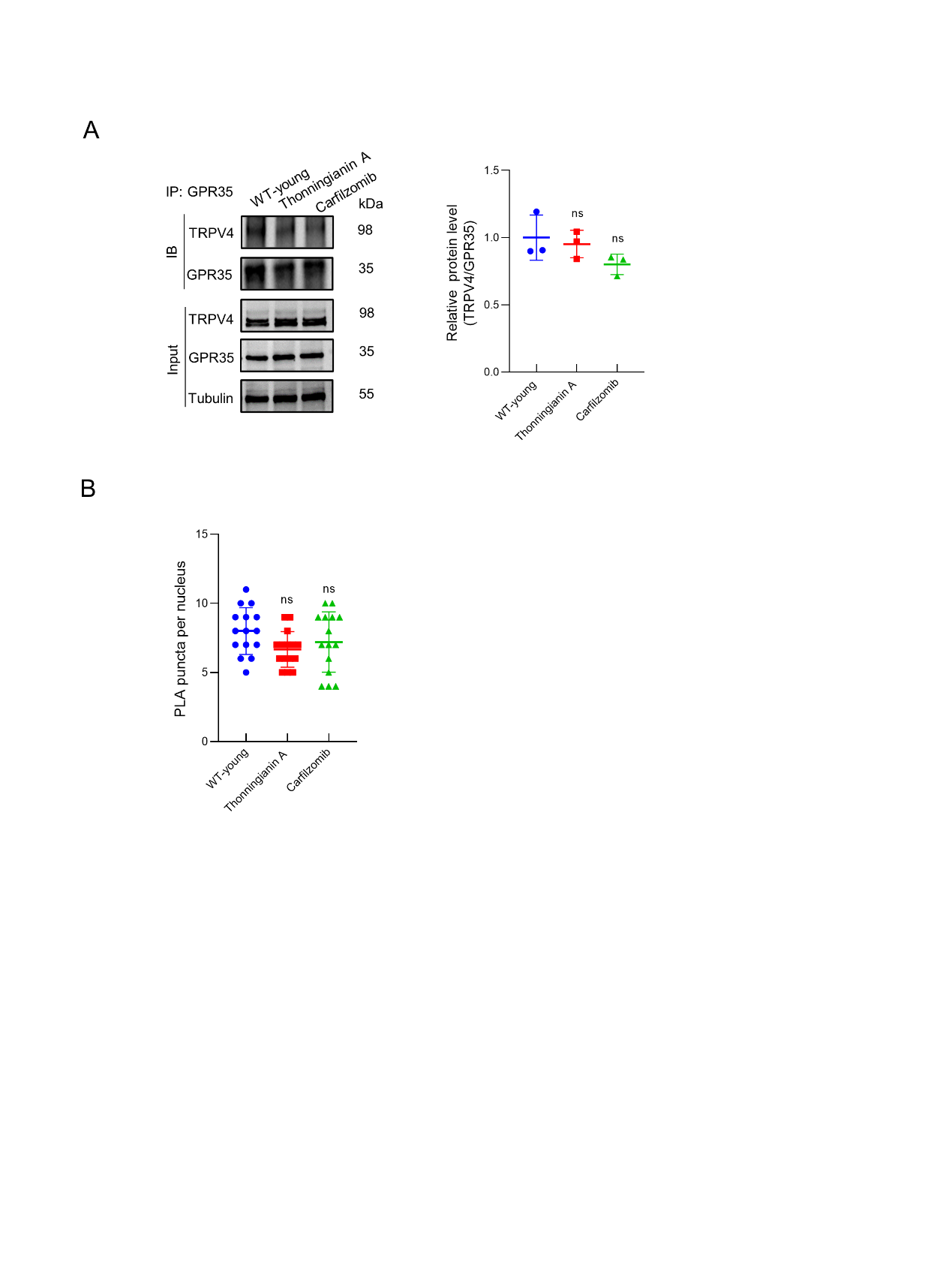
Figure S8：Thonningianin A and Carfilzomib did not significantly suppress the TRPV4-GPR35 interaction in WT-young primary ECs. (A) Co-immunoprecipitation (co-IP) assay of TRPV4 and GPR35 in primary mesenteric ECs from WT-young mice incubated with Thonningianin A (10 μM) and Carfilzomib (1 μM) for 30 minutes before harvest. The lysates were immunoprecipitated with control IgG or GPR35 antibody, followed by immunoblotting (IB) with TRPV4 antibody. The quantitative analysis is shown on the right, n=3 isolations for each group; ns indicates no statistical significance vs. WT-young ECs, one-way ANOVA. (B) Quantification of TRPV4_EC_-GPR35_EC_ co-localization on WT-young primary ECs treated with Thonningianin A (10 μM) and Carfilzomib (1 μM), detected by PLA assays (n = 15 cells from 4-5 isolations for each group; ns indicates no statistical significance vs. WT-young ECs, one-way ANOVA).


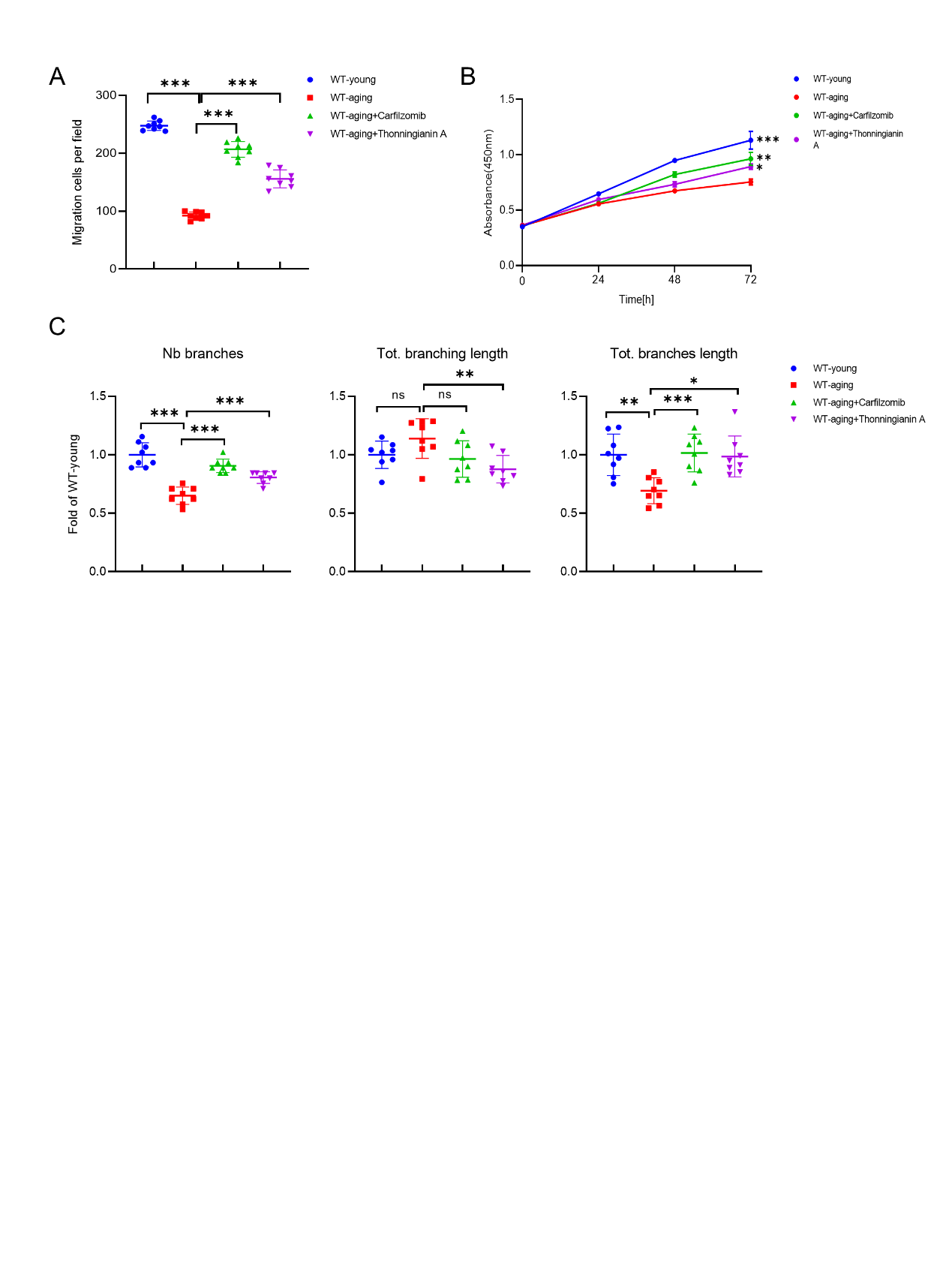
Figure S9: Effects of Carfilzomib and Thonningianin A on the regulation of functions in aging ECs. (A) Comparison of migrated cells per field among primary ECs from WT-young, WT aging, and WT-aging treated with Carfilzomib or Thonningianin (n=8 for each group; ***p<0.001 vs. WT-aging; one-way ANOVA). (B) Cell viability of ECs assessed by CCK-8. Various treatments were identical to those listed in (A) (n=3 for each group; *p<0.05 vs. WT aging, **p<0.01 vs. WT aging, ***p<0.001 vs. WT aging; two-way ANOVA). (C) Comparison of EC tube formation ability assessed under the same conditions as listed in (A) (n=8 for each group; **p<0.01 vs. WT aging, ***p<0.001 vs. WT aging; one-way ANOVA).

**Appendix**

All exact p values for the comparisons presented in the figures are provided below.

|  | p value | Summary |
| --- | --- | --- |
| Figure 1B (two-way ANNOVA) | | |
| WT-young vs. WT-aging | 0.000030 | *** |
| WT-young vs. TRPV4_EC_^-/-^-young | <0.000001 | *** |
| TRPV4_EC_^-/-^-young vs. TRPV4_EC_^-/-^-aging | 0.550427 | ns |
| Figure 1C (one-way ANNOVA) | | |
| WT-young vs. WT-aging | <0.000001 | *** |
| WT-young vs. TRPV4_EC_^-/-^-young | <0.000001 | *** |
| Figure 1E (t-test) | | |
| WT-young vs. WT-aging | 0.000145 | *** |
| Figure 2B (two-way ANNOVA) | | |
| WT-young vs. TRPV4_EC_^-/-^-young | 0.494928 | ns |
| WT-young vs. WT-aging | 0.006465 | ** |
| WT-aging vs. TRPV4_EC_^-/-^ -aging | 0.020600 | * |
| Figure 2C (two-way ANNOVA) | | |
| WT young vs. TRPV4_EC_^-/-^ young | >0.99999 | ns |
| WT-young vs. WT-aging | 0.000884 | *** |
| WT-aging vs. TRPV4_EC_^-/-^ -aging | 0.001188 | ** |
| Figure 2E (one-way ANNOVA) | | |
| WT-young vs. WT-aging | <0.000001 | *** |
| WT-young vs. TRPV4_EC_^-/-^ | <0.000001 | *** |
| WT-young vs. AAV-FLT1-shRNA (GPR35) | <0.000001 | *** |
| Figure 2F (t-test) | | |
| WT-young vs. WT-aging | 0.025305 | * |
| Figure 3A (one-way ANNOVA) | | |
| Control vs. siGPR35 | 0.001389 | ** |
| Control vs. GSK101 | <0.000001 | *** |
| Control vs. HC0+OEGPR35 | <0.000001 | *** |
| Control vs. GSK101+OEGPR35 | <0.000001 | *** |
| Control vs. GSK101+siGPR35 | <0.000001 | *** |
| Figure 3B (Nb branches/field) (one-way ANNOVA) | | |
| Control vs. siGPR35 | 0.006263 | ** |
| Control vs. GSK101 | 0.000345 | *** |
| Control vs. HC0+OEGPR35 | 0.016164 | * |
| Control vs. GSK101+OEGPR35 | 0.012152 | * |
| Control vs. GSK101+siGPR35 | 0.000269 | *** |
| Figure 3B (Tot. branching length/field) (one-way ANNOVA) | | |
| Control vs. siGPR35 | 0.999747 | ns |
| Control vs. GSK101 | 0.861866 | ns |
| Control vs. HC0+OEGPR35 | <0.000001 | *** |
| Control vs. GSK101+OEGPR35 | <0.000001 | *** |
| Control vs. GSK101+siGPR35 | 0.592023 | ns |
| Figure 3B (Tot. branches length/field) (one-way ANNOVA) | | |
| Control vs. siGPR35 | 0.178775 | ns |
| Control vs. GSK101 | <0.000001 | *** |
| Control vs. HC0+OEGPR35 | 0.025699 | * |
| Control vs. GSK101+OEGPR35 | 0.004936 | ** |
| Control vs. GSK101+siGPR35 | 0.176212 | ns |
| Figure 3C (one-way ANNOVA) | | |
| Control vs. siGPR35 | 0.003820 | ** |
| Control vs. GSK101 | 0.004508 | ** |
| Control vs. HC0+OEGPR35 | 0.000040 | *** |
| Control vs. GSK101+OEGPR35 | 0.000131 | *** |
| Control vs. GSK101+siGPR35 | 0.002152 | ** |
| Figure 4 A (t-test) | | |
| WT vs. TRPV4_EC_^-/-^ | <0.000001 | *** |
| Figure 4 B (t-test) | | |
| Zap vs. Zap+HC0 | 0.000055 | *** |
| GSK101 vs. AAV-FLT1-shRNA (GPR35)  +GSK101 | 0.106049 | ns |
| Figure 4 C (two-way ANNOVA) | | |
| Control vs. HC0 | <0.000001 | *** |
| Control vs. TRPV4_EC_^-/-^ | <0.000001 | *** |
| Figure 4D (two-way ANNOVA) | | |
| AAV-FLT1-shRNA (NC) vs. AAV-FLT1-shRNA (GPR35) | 0.610606 | ns |
| Figure 5A (one-way ANNOVA) | | |
| WT-aging vs. Thonningianin A | 0.003422 | ** |
| WT-aging vs. Carfilzomib | <0.000001 | *** |
| WT-aging vs. Naringin | 0.463630 | ns |
| WT-aging vs. Diosmin | 0.836786 | ns |
| WT-aging vs. Navitoclax | 0.977718 | ns |
| Figure 5B (one-way ANNOVA) | | |
| WT-aging vs. Thonningianin A | <0.000001 | *** |
| WT-aging vs. Carfilzomib | <0.000001 | *** |
| Figure 5C left (two-way ANNOVA) | | |
| WT-aging vs. WT-aging + Carfilzomib | 0.022068 | * |
| WT-aging vs. AAV-FLT1-shRNA (GPR35)-aging+ Carfilzomib | 0.306069 | ns |
| Figure 5C right (two-way ANNOVA) | | |
| WT-aging vs. WT-aging + Carfilzomib | 0.007560 | ** |
| WT-aging vs. TRPV4_EC_^-/-^-aging+ Carfilzomib | 0.023068 | * |
| Figure 5D left (two-way ANNOVA) | | |
| WT-aging vs. WT-aging + Carfilzomib | 0.026798 | * |
| WT-aging vs. AAV-FLT1-shRNA (GPR35)-aging+ Carfilzomib | 0.731948 | ns |
| Figure 5D right (two-way ANNOVA) | | |
| WT-aging vs. WT-aging + Carfilzomib | 0.013431 | * |
| WT-aging vs. TRPV4_EC_^-/-^-aging+ Carfilzomib | 0.002407 | ** |
| Figure 5E (one-way ANNOVA) | | |
| WT-aging vs. WT-young | 0.000254 | *** |
| WT-aging vs. WT-aging+Carfilzomib | <0.000001 | *** |
| WT-aging vs. WT-aging+Thonningianin A | 0.002167 | ** |
| WT-aging vs. TRPV4_EC_^-/-^ | <0.000001 | *** |
| Figure 5F (one-way ANNOVA) | | |
| WT-aging vs. WT-young | <0.000001 | *** |
| WT-aging vs. WT-aging+Carfilzomib | <0.000001 | *** |
| WT-aging vs. WT-aging+Thonningianin A | 0.018148 | * |
| WT-aging vs. TRPV4_EC_^-/-^ | <0.000001 | *** |
| Figure S1 (one-way ANNOVA) | | |
| Control vs. siGPR35-1 | <0.000001 | *** |
| Control vs. siGPR35-2 | 0.016286 | * |
| Control vs. siGPR35-3 | 0.002350 | ** |
| Figure S3A (one-way ANNOVA) | | |
| WT-young vs. WT-aging | >0.999999 | ns |
| WT-young vs. TRPV4_EC_^-/-^ | <0.000001 | *** |
| Figure S3B (t-test) | | |
| Young vs. Aging | 0.373187 | ns |
| Figure S5A (t-test) | | |
| WT vs. TRPV4_EC_^-/-^ | <0.000001 | *** |
| Figure S5B (two-way ANNOVA) | | |
| WT vs. TRPV4_EC_^-/-^ | <0.000001 | *** |
| Figure S6A (t-test) | | |
| AAV-FLT1-shRNA (NC) vs. AAV-FLT1-shRNA (GPR35) | <0.000001 | *** |
| Figure S8A (one-way ANNOVA) | | |
| WT-young vs. Thonningianin A | 0.850028 | ns |
| WT-young vs. Carfilzomib | 0.156380 | ns |
| Figure S8B (one-way ANNOVA) | | |
| WT-young vs. Thonningianin A | 0.079491 | ns |
| WT-young vs. Carfilzomib | 0.361234 | ns |
| Figure S9A (one-way ANNOVA) | | |
| WT-aging vs. WT-young | <0.000001 | *** |
| WT-aging vs. WT-aging+Carfilzomib | <0.000001 | *** |
| WT-aging vs. WT-aging+Thonningianin A | <0.000001 | *** |
| Figure S9B (two-way ANNOVA) | | |
| WT-aging vs. WT-young | 0.000186 | *** |
| WT-aging vs. WT-aging+Carfilzomib | 0.004162 | ** |
| WT-aging vs. WT-aging+Thonningianin A | 0.028018 | * |
| Figure S9C (Nb branches) (one-way ANNOVA) | | |
| WT-aging vs. WT-young | <0.0001 | *** |
| WT-aging vs. WT-aging+Carfilzomib | <0.0001 | *** |
| WT-aging vs. WT-aging+Thonningianin A | 0.000766 | *** |
| Figure S9C (Tot. branching length) (one-way ANNOVA) | | |
| WT-aging vs. WT-young | 0.147653 | ns |
| WT-aging vs. WT-aging+Carfilzomib | 0.053861 | ns |
| WT-aging vs. WT-aging+Thonningianin A | 0.002697 | ** |
| Figure S9C (Tot. branching length) (one-way ANNOVA) | | |
| WT-aging vs. WT-young | 0.001622 | ** |
| WT-aging vs. WT-aging+Carfilzomib | 0.000975 | *** |
| WT-aging vs. WT-aging+Thonningianin A | 0.002617 | ** |
